# Availability of Access, Watch, and Reserve groups of essential antibiotics: a cross-sectional survey

**DOI:** 10.3389/fpubh.2023.1251434

**Published:** 2024-01-04

**Authors:** Sunaina Rafi, Syed Muneeb Anjum, Muhammad Usman, Hafiz Awais Nawaz, Mamoona Chaudhry, Zaheer-Ud-Din Babar, Huma Rasheed

**Affiliations:** ^1^Institute of Pharmaceutical Sciences, University of Veterinary and Animal Sciences, Lahore, Pakistan; ^2^Department of Epidemiology and Public Health, University of Veterinary and Animal Sciences, Lahore, Pakistan; ^3^Department of Pharmacy, University of Huddersfield, Huddersfield, United Kingdom

**Keywords:** essential antibiotics, AWaRe, availability, public and private health sector, Access, essential medicines, antimicrobial resistance, rational use of antibiotics

## Abstract

**Background:**

Lower-middle income countries face drastic challenges in Access to essential medicines. Data regarding Pakistan is scarce with no comprehensive study in this regard. The objectives of the study are to document and compare public and private sector availability of all essential antibiotics as well as to conduct a comparison among the AWaRe groups.

**Methods:**

The study analyzed 103 essential antibiotics comprising 51 Access, 29 Watch, 6 Reserve, and 17 anti-tuberculosis drugs from 15^th^ August to 10^th^ September 2020 in Lahore, Pakistan. It included on-spot physical availability and availability trend surveys. The survey sites included five public tertiary care hospitals with one as anchor and four randomly selected. Their hospital pharmacies and one randomly selected private retail pharmacy from the vicinity each hospital comprised the ten sampling sites. Percentage availability for each antibiotic was categorized as high (>80%), fairly high (50–80%), low (30–<50%), very low (*<*30–>0%), and not available (0%).

**Results:**

The mean percentage on-spot availability was 23.76% ± 5.19 (14–25%) for public facilities and 59.20% ± 4.45 (54–66%) for private sector retail pharmacies. The overall percentage of available essential antibiotics varied significantly (p** < 0.001) in public and private sector sampling sites. Except for the Watch group, all other groups showed the mode of 0% availability. A significant difference (p**** < 0.00001) was seen in percentage availability by Access, Watch, Reserve, and anti-TB-all groups of essential antibiotics. The availability trend survey revealed a list of 18 medicines as ‘as never been available’, and five medicines were ‘not available for 5 years or more than 5 years.’ Fourteen medicines as ‘never been heard.’

**Conclusion:**

Non-availability of essential medicines is a significant public health challenge at public-sector facilities in Pakistan. It was observed that a number of essential antibiotics were not available in both public and private sectors. A number of corrective strategies are required. This includes the engagement of stakeholder and government bodies. This can help to improve supply chain barriers.

## Introduction

Timely, equitable, and unhindered Access to quality medicines is included in the human right to health (1). Access to effective antimicrobials is a worldwide challenge, and non-availability and delays in Access to antibiotics kill more people than resistance to antibiotics (2). More than a million children die each year due to untreated pneumonia and sepsis, and at the same time, the medicines are also losing their effectiveness owing to growing resistance (2).

Improvements in Access to antimicrobials and life probability are also documented in recent years, especially in low-income and lower-middle-income countries (2). Improvement in availability of penicillin resulted in lowering of mortality rate of pneumococcal pneumonia (20–40% to 5%) and pneumococcal bacteremia (50–80% to 18–20%) (2). Antibiotics have been used not only for infectious diseases but also to support modern medical care, particularly in the ability to perform surgeries, organ replacements, and treatment of cancers (2).

Introduced in 2017, the World Health Organization classified the essential antibiotics into AWaRe groups named as Access, Watch, and Reserve (3). The purpose of this classification was to control antimicrobial resistance globally and to develop a tool for national antibiotic stewardship programs (4). This classification provides a dynamic tool for appropriate use of antibiotics at global, national, and local levels (5).

**Access** group antibiotics have the lowest resistance potential than the antibiotics in other groups and are high-priority antibiotics for availability, quality assurance, and affordability to both individuals and the community (5). Contrary to that, **Watch** group antibiotics have higher resistance potential but are still a significant medicine for Access, and these medicines are ranked as key targets of stewardship programs and monitoring (5).

Critically important antibiotics and antibiotic classes that should be reserved for treatment of confirmed or suspected infections due to multi-drug resistant (MDR) organisms are included in the third and most closely monitored group called the “**Reserve**” group. Antibiotics included in the Reserve group are classed as “last resort” options (5). These antibiotics should be accessible to highly specific patients and settings only when all substitutes have been botched or are not suitable. To preserve their effectiveness, these medicines should be protected and ranked as key targets of national and international stewardship programs that involve utilization reporting and monitoring (6).

The AWaRe classifications are under constant revision and closely underplay the guidelines for rationalizing antimicrobial consumption. Indicators such as Access-to-Watch ratio and percentage of amoxicillin consumption have been devised as instruments to gauge the rational use of antibiotics (7, 8). Increasing the ratio of Access group antibiotics to 60% of total antibiotic consumption is set as a country-level target by WHO 13th General Programme of Work (2019–2023) (3), which demands documentation and improvement in the availability for gap areas among this group of antibiotics.

The AWaRe classification has been revised in 2021 with the issuance of the 22nd WHO Model Essential Medicines List (3).

Anti-infectives form the largest therapeutic category of 22nd WHO model EML and the National Essential Medicines List of Pakistan issued in 2018 (NEMLPK-2018). “Antibacterials” are present in the sub-category 6.2 and are presented according to AWaRe classification in both lists (9).

Pakistan is a low-middle-income country with a significant burden of infectious diseases along with a growing number of non-communicable diseases. Non-availability of essential medicines from the Access group such as phenoxymethyl penicillin, cloxacillin/flucloxacillin, benzyl penicillin, and benzathine penicillin results in the absence of medicines of first choice causing cost implications as well as development of resistance due to lesser choices of medicine for a given antimicrobial spectrum as well as compromised disease prognosis. There should be restrictions on the Watch and Reserve medicine use, and surveillance mechanisms should be developed to record and control consumption patterns. The study provides evidence for clear policy decisions. In Pakistan, the irrational use of antibiotics is widespread consisting of inappropriate use, self-medication, and overuse. There are reports of non-availability of antibiotics in public and private sector health facilities. However, studies documenting the non-availability of antimicrobials, particularly antibiotics for LMICs show that the “global antibiotics supply is patchy, complex, and at risk of collapsing” (10). The data regarding Pakistan are scarce, and there is no comprehensive study based on a field survey documenting the on-spot availability of the entire range of essential antibiotics included in NEML 2018.

The study was conducted in Lahore, the second largest city in Pakistan. It has a population of 11.126285 million people and covers 1,772 km^2^ area (11). The study methodology was adapted from the protocol by Health Action International (HAI) and World Health Organization (WHO) (12) with the objectives to document and compare the availability of essential antibiotics in the city of Lahore in public and private sectors as well as to conduct group comparison for the availability of essential antibiotics categorized according to AWaRe classification.

## Methods

A cross-sectional field study for the availability of essential antibiotics was conducted using the adaptation of WHO/HAI methodology (12). It was conducted from 15th August to 10th September 2020 in public and private sector pharmacies in Lahore, Pakistan. The survey included the on-spot physical availability check for essential antibiotics as well as documentation of availability trends.

### Consent and confidentiality

The pharmacy personnel at the randomly selected survey sites and the study anchor were approached in person during the study period and briefed on the study objectives and confidentiality statement (Annexure A). They were requested for written consent after provision of which the survey was started using a coded pre-designed survey form (Annexure B) without any identity of the respondents. The survey was conducted among the pharmacy staff, and no minors were involved in it. Both respondents and the survey sites were kept anonymous using coding, and no one had Access to the identity of respondents except the interviewer.

### Selection of essential antibiotics for inclusion in the study

The study was conducted to check the physical availability of 103 registered essential antibiotics extracted from NEML-PK-2018 as enlisted in the previously published study (13). The number of antibiotics covered in the study from each group of Access (beta lactam + others), Watch (beta lactam + others), Reserve, and antituberculosis medicines (anti-TB without duplicates) was 28 + 23, 12 + 17, 6, and 17, respectively, using the categorization provided by NEML 2018 for AWaRe classification of antibiotics. The antituberculosis medicines without duplicates did not include duplicates, i.e., the antibiotics that are used in the treatment of tuberculosis as well as other bacterial infections, and they also fall under one of the AWaRe groups in accordance with the AWaRe Classification provided by WHO. A similar approach for the selection of antibiotics was also used in a study conducted in Bangladesh in which they selected the list of essential antibiotics from their national essential medicines list to measure the availability and affordability of antibiotics (14).

### Ethical approval

The study was approved by the Institutional Review Committee for Biomedical Research (IRC/BMR) vide letter No. 083/IRC/BMR (a).

### Sampling, survey sites, and data collection

As this survey was conducted during the COVID-19 pandemic, the survey period was kept shorter. One public-sector hospital and the private drug sale outlet in the surrounding of this public hospital were covered in 1 day (that means two survey sites were covered in 1 day). Before going into the main survey, a pilot survey was conducted on a random private drug sale outlet to practice the adopted methodology.

Two sectors including the public health sector hospitals and the private sector retail pharmacies were covered in the surveys. The list containing 19 tertiary care hospitals was retrieved from the official website of Punjab Government (15). Six tertiary care hospitals for specialized care were excluded. Out of the remaining 13 hospitals, one hospital was designated as the main hospital (or anchor site), and randomization was performed on the rest of the 12 public-sector tertiary care hospitals using an online randomization tool (16) to randomly select four additional hospitals for inclusion in the survey in accordance with the HAI/WHO methodology (12).

Similarly, the private sector retail pharmacies (including the medical stores) within the vicinity of the five selected hospitals were enlisted, and one facility was identified through random selection for collection of data (Supplementary Figure S1). Two backup random selections were also maintained for use in case of refusal for participation in the study. The exclusion criteria of medicines stocks less than 50% was not included in the protocol as the pilot study showed less than 50% availability. The public sector was represented by alphabet “A,” and the private sector by “B.” The digit (1–5) in the prefix showed the enumeration of the survey site.

### Training for data collection

Interviewers were trained using an interview guide (Annexure-D) for the data collection process, and consent was taken using a consent form (Annexure A). Interviewers were briefed and cross-questioned to confirm the understanding of survey forms and survey guide. Data collection on availability was done using pre-designed Performa (Annexure B) which was tested on a pilot site for face validation by interviewing a pharmacy staff. A short demographic detail of the survey site excluding name, identity of the premises, and the respondents was also collected from each sampling site (Annexure C).

### Data collection

Two availability surveys were conducted:

#### Survey 1: physical availability check for antibiotics

The survey was performed using the pre-designed Performa (Annexure B) to record the on-spot physical availability of essential antibiotics at public hospitals and at private sector retail pharmacies. The brand names and registration numbers were noted for antibiotics, which were available on the site of data collection.

Data entry on the Performa (Annexure B) was done for the on-spot physical availability and the responses were categorized as “available,” “not available,” and “available in slightly different specifications.” This was done by using a numeric entry of “1,” “2,” and “3,” respectively, for these responses and the same was entered into an MS Excel worksheet. Color codes of “red,” “green,” and “orange” were used in MS Excel sheet to represent available, not available, and slightly different specifications, respectively.

The term “available” meant that the medicinal product was present physically at the premises at the time of the survey, whereas “not available” referred that the medicinal product was not present physically at the premises at the time of survey, and “available in slightly different specification” was the third response showing that the essential medicine was available on spot with a slight difference in the specification. The difference in specification was noted.

Percentage availability was calculated for each antibiotic included in the survey using the following formula:

$$\mathrm{Percentage}\;\mathrm{availability}=\left(\begin{array}{l} \mathrm{Number}\;\mathrm{of}\;\mathrm{survey}\;\mathrm{sites}\;\mathrm{where}\;\\ \mathrm{medicine}\;\mathrm{was}\;\mathrm{found}\;\mathrm{available}\;\\ \mathrm{on}\;\mathrm{the}\;\mathrm{spot}/\mathrm{Total}\;\mathrm{number}\;\mathrm{of}\;\\ \mathrm{survey}\;\mathrm{sites} \end{array}\right)\times 100.$$

Antibiotics were categorized as high, fairly high, low, very low, and not available adapted from WHO thresholds (5) at >80% (purple), 50–80% (blue), 30–<50% (orange), <30–>0% (yellow), and 0% (red), respectively, on the basis of the calculated percentage availability. The same approach as percentage availability has also been used in another study in China (17).

#### Survey 2: documentation of availability trend

The pre-designed Performa (Annexure B) also constituted survey questions to document the three availability trends: available, short supply (SS), and not available.

**Available:** The term “Available (A)” meant that antibiotics were present on the spot, and there was no history of any issue regarding the availability (shortages or not-availability) experienced in the last 1 year for that antibiotic.

**Short supply:** The term short “supply (SS)” meant that antibiotic was available on and off in the last 1 year or was available at the spot but was in short supply and could have been present but in quantities insufficient to cater to the current supply demand.

**Not available:** The term “not available (NA)” meant that the antibiotic was not available, and the time since it was not available was selected from the five options (last 1 year, last 2 years, last 5 years, for more than 5 years, or never been available).

Respondents could also add remarks to this survey portion and the remarks included do not know, not in demand, available on demand (details of supplier were inquired in this case), available in different specifications from the enlisted one (available specifications were noted down), or any incidence or information relating to Access about this antibiotic.

### Statistical analysis

Statistical analysis was performed for each group of Access, Watch, anti-TB, and Reserve groups of essential antibiotics based on the percentage availability recorded from public-sector hospitals and at private sector retail pharmacies using the Mann–Whitney U-test (*p* < 0.05 with 95% CI). Group comparison of percentage availability of Access, Watch, Reserve, and anti-TB medicines without duplicates, and anti-TB-all groups of essential antibiotics were performed using the Kruskal–Wallis (post-hoc Dunn) ANOVA test (*p* < 0.05 with 95% CI). All statistical tests were performed using GraphPad Prism version 6.0.1 for Windows (18). Descriptive statistics and color-coded heat maps were used to illustrate the gaps and trends in data for comparison among the three groups of antibiotics. Mean, mode, and median of the percentage availability were also calculated for each antibiotic group of Access, Watch, and Reserve.

## Results

### Availability of essential antibiotics

The field survey to document the on-spot physical availability of essential antibiotics was conducted at public and private sector retail pharmacies along with the documentation of the availability trends. The subcategory 6.2 of antibiotics in NEMLPK-2018 (9) consisted of 53 generics and 136 essential medicinal products; out of which, 45 generics and 103 essential medicinal products were found registered in a survey in the previous study (13) and are included in this study.

The survey sites including five hospital pharmacies of public-sector tertiary care hospitals and five private sector retail pharmacies were selected for inclusion in the study. The respondents of the public hospitals provided consent, whereas one private retail pharmacy did not provide consent, so the next site in the randomization was opted. One private retail pharmacy was excluded because of closing of business. The demographics of respondents and facilities information are given in Supplementary Table S1.

### Survey 1 results: on-spot physical availability check

The exact number of antibiotics from the surveyed 103 antibiotics that were found available at each sampling site is shown in Supplementary Tables S2, S3. Supplementary Table S2 shows the availability of 103 antibiotics with the mean of 23.76 ± 5.19 for public facilities and mean of 59.20 ± 4.45 for private sector retail pharmacies. The Mann–Whitney unpaired test with 95% CI supports that the overall percentage of available essential antibiotics varied significantly in public and private sector sampling sites (p** < 0.001) (Supplementary Figure S2). The same results were shown for each group, i.e., Access, Watch, and Reserve group of antibiotics (p** < 0.001), except for the list of 17 anti-TB medicines without duplicates (Supplementary Figure S3). Their availability was consistently low in both public and private sectors.

#### Group analysis

##### Heat map

The heat maps for Access (beta lactam and other than beta lactam), Watch (beta lactam and other than beta lactam), Reserve, and all the essential anti-tuberculous medicines (Supplementary Tables S4–S9) show distribution of on-spot availability of essential antibiotics using the color codes of red (not available), green (available), and orange (available with slightly different specification).

In total, six products varied in their product specifications from the essential antibiotics list used in the survey, three being pediatric suspensions, i.e., one injectable and two anti-TB fixed-dose combinations (Table 1).

**Table 1:** Medicinal products available at a tertiary care hospital with slightly different specifications.

Medicinal product enlisted in NEML				Medicinal products with slightly different specifications		
1	Amoxicillin+ clavulanic acid	Oral liquid	250 mg + 62.5 mg	Amoxicillin+ clavulanic acid	Oral liquid	457 mg
2	Meropenem	Injection	500 mg	Meropenem	Injection	1 g
3	Azithromycin	Oral liquid	200 mg/5 mL	Azithromycin	Oral liquid	100 mg/5 mL
4	Clarithromycin	Oral liquid	125 mg/5 mL	Clarithromycin	Oral liquid	250 mg/5 mL
5	Ethambutol+ isoniazid+ rifampicin	Tablet	275 mg+ 75 mg+ 150 mg	Ethambutol+ isoniazid+ rifampicin	Tablet	300 mg+ 75 mg+ 150 mg
6	Isoniazid+ pyrazinamide+ rifampicin	Tablet	75 mg+ 400 mg+ 150 mg	Isoniazid+ pyrazinamide+ rifampicin	Tablet	50 mg+ 150 mg+ 50 mg

#### Percentage availability for each antibiotic

Percentage availability was calculated, and color was coded for categorization, as stated in the section Methods [high (>80%) as purple, fairly high (50–80%) as blue, low (30–*<*50%) *as* yellow, very low (*<*30–>0%) as orange, and not available (0%) as red] for each essential antibiotic (Supplementary Tables S5–S10) presented in the last column of the table. The distribution of antibiotics in each category in accordance with the percentage availability documented in the study is shown in Supplementary Figure S4.

The mean, median, mode, and range (minima and maxima) of each group are shown in Table 2. Except for the Watch group, all the groups showed the mode of 0% availability. The median was 0%, 10%, 40%, 50%, and 70% for anti-TB medicines without duplicates and anti-TB (all) Access, Reserve, and Watch groups, respectively (Table 2).

**Table 2:** On-spot percentage availability of essential antibiotic in accordance with the aWaRe classification.

Percentage availability (%)	Anti-TB without duplicates	Anti TB all*	Access	Reserve	Watch
Number of antibiotics	17	28	51	6	29
Average	10.59	28.21	37.65	43.33	64.83
Min	0.00	0.00	0.00	0.00	0.00
Max	70.00	90.00	100.00	90.00	100.00
Mode	0.00	0.00	0.00	0.00	90.00
Median	0.00	10.00	40.00	50.00	70.00

*Including antibiotics from Access, Watch, or Reserve classes duplicated in anti-TB medicines.

**Figure 1:**
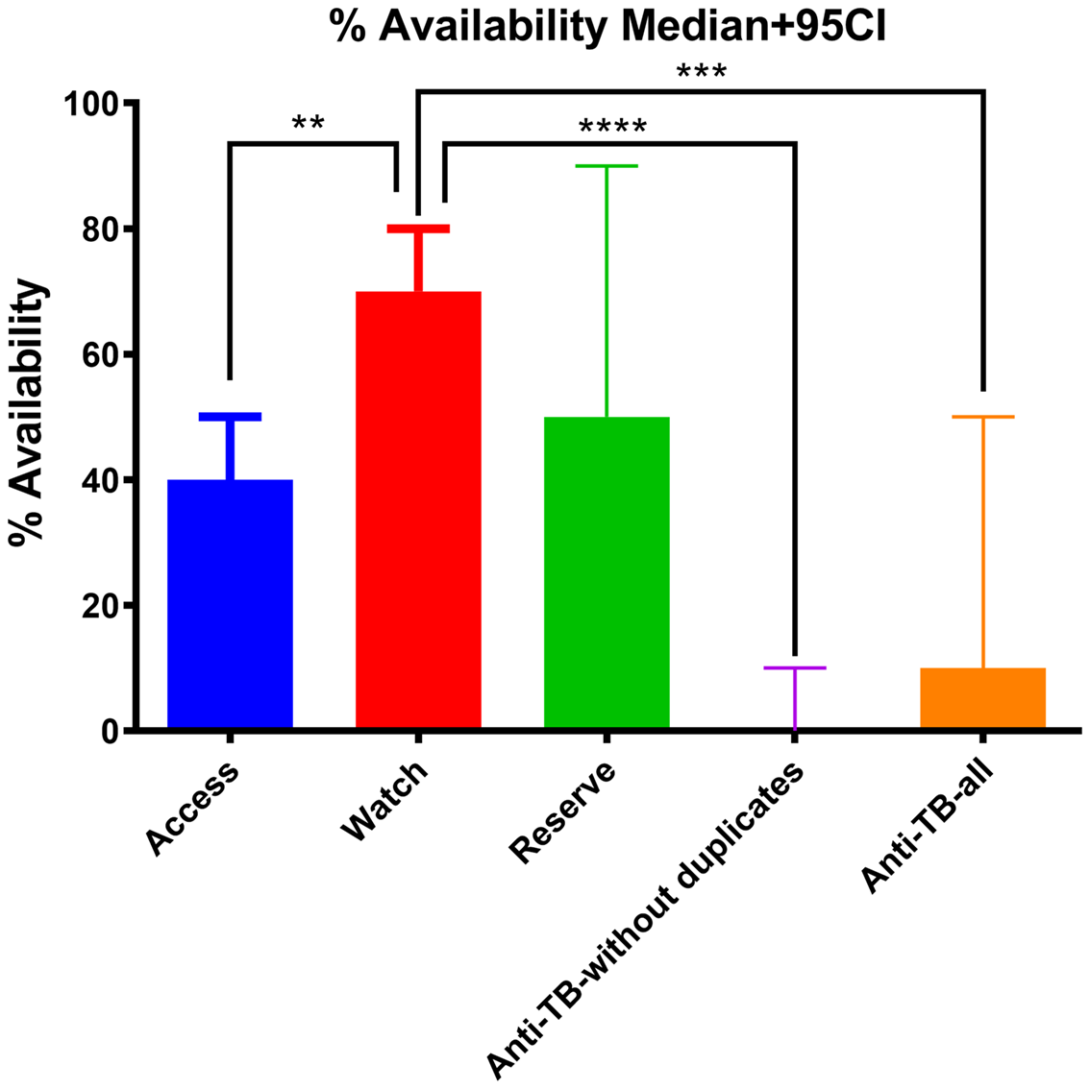
Group comparison of percentage availability of Access, Watch, Reserve, and anti-TB without duplicates, and anti-TB all groups of essential antibiotics using the Kruskal–Wallis (post-hoc Dunn) ANOVA test (p**** < 0.00001 with 95% CI).

Group comparison of percentage availability of Access, Watch, Reserve, and anti-TB medicines without duplicates and anti-TB all groups of essential antibiotics using the Kruskal–Wallis ANOVA test (p**** < 0.00001 with 95% CI) is shown in Figure 1 with a median of <0.05. Similarly, Table 3 shows the multiple comparison using Dunn’s multiple comparison test with the results being fairly significant (*p* < 0.001) for the Watch–Access pair, very highly significant (*p* < 0.00001) for the Watch anti-TB without duplicates pair, and highly significant (*p* < 0.0001) for the Watch anti-TB (all) pair. The variations between the results of other group pairs were not found significant.

**Table 3:** Results of multiple comparison of Access, Watch, Reserve, anti-TB W&R, and anti-TB (all anti-TB medicines) using Dunn’s multiple comparison test with 95% CI.

Dunn”s multiple comparisons test	Summary	Adjusted *p*-value	Pair
Access vs. Watch	**	0.003614	A-B
Access vs. Reserve	ns	>0.999999	A-C
Access vs. Anti-TB-without duplicates	ns	0.060689	A-D
Access vs. Anti-TB-all	ns	>0.999999	A-E
Watch vs. Reserve	ns	>0.999999	B-C
Watch vs. Anti-TB-without duplicates	****	0.000002	B-D
Watch vs. Anti-TB-all	***	0.000631	B-E
Reserve vs. Anti-TB-without duplicates	ns	0.534741	C-D
Reserve vs. Anti-TB	ns	>0.999999	C-E
Anti-TB-without duplicates vs. Anti-TB-all	ns	0.801483	D-E

ns, non-significant; *significant; **significance; ***highly significant; ****very highly significant; A, Access; B, Watch; C, Reserve; D, Anti-TB-without duplicates; E, Anti-TB-all.

**Figure 2:**
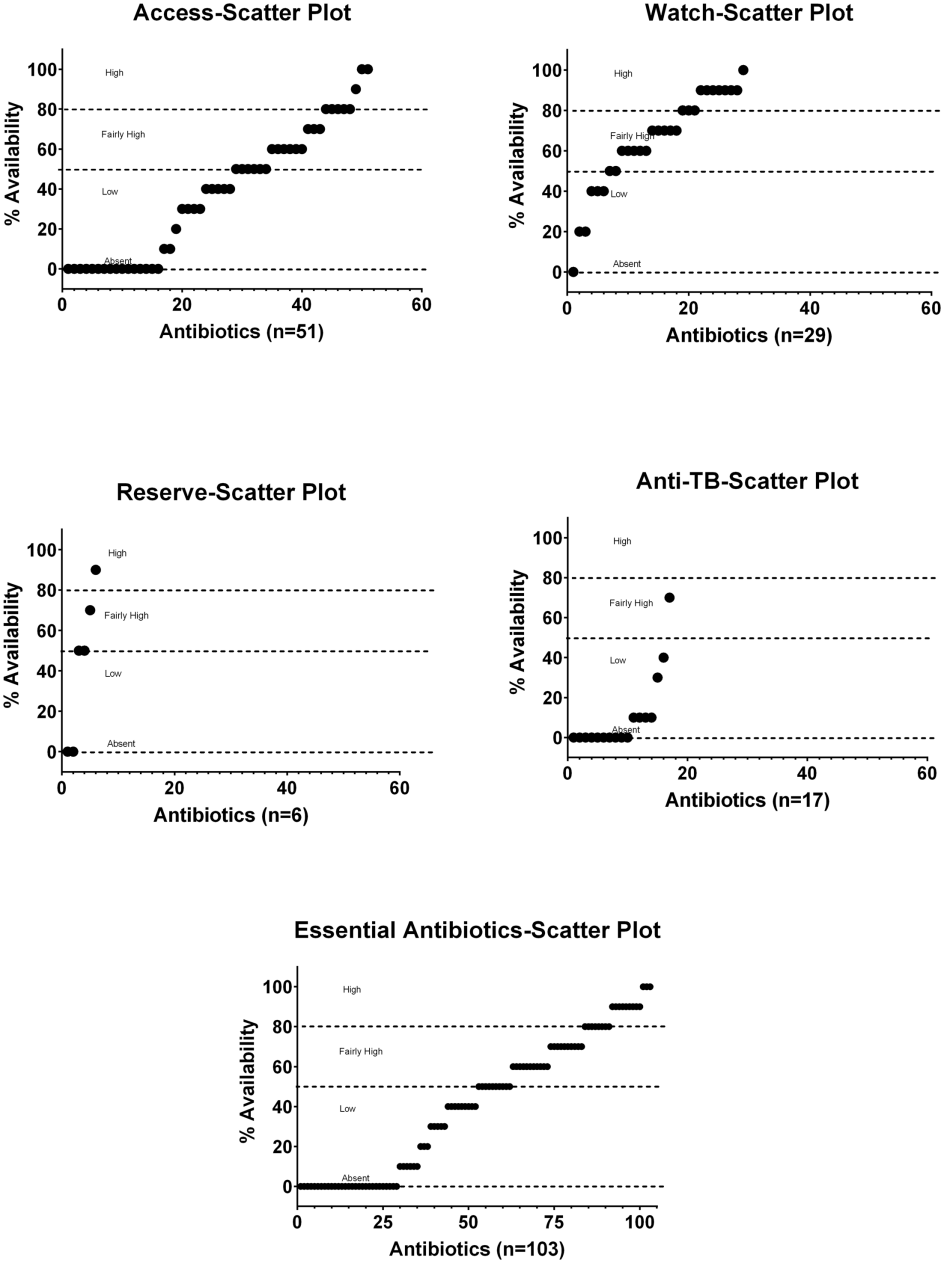
Scatter plots of Access, Watch, Reserve, and anti-TB without duplicates groups of essential antibiotics for the percentage of age availability.

Figure 2 shows scatter plots of Access, Watch, Reserve, and anti-TB medicines without duplicate groups of essential antibiotics for the percentage availability and covers the overall distribution of the surveyed products for their on-spot availability pattern.

The complete descriptive summary with respect to availability status at sampling sites as a percentage of the total surveyed antibiotics (*n* = 103) is shown in Supplementary Table S10. It is shown that only 2.91% of the surveyed essential antibiotics were available at all survey sites, whereas 30.9% antibiotics were not available at any survey site. The details of antibiotics that showed a positive availability pattern with respect to all the surveyed sites or to public or private facility as well as the antibiotics that showed non-availability at all the survey sites or limited availability are listed in Supplementary Figure S5 using yellow and blue text boxes, respectively. Tab. co-amoxiclav 625 mg, Inj. ceftriaxone 1 g, and Inj. metronidazole 500 mg/100 mL were available at all survey sites, 42 antibiotics were available at all private sector retail pharmacies, 10 antibiotics were not available at one hospital, and 13 antibiotics were not available at one private sector retail pharmacy. Similarly, the four fixed-dose combinations for anti-TB medicines without duplicates were not available at any private sector retail pharmacy. There were 31 antibiotics that were not present at any survey site, 22 essential antibiotics were not available at any hospital, and 19 essential antibiotics were available only at just one public facility (Supplementary Figure S5).

#### Physical availability of essential antibiotic (key Access group) at survey sites

Unlike the Watch group where none of the antibiotics were not available at any sites except moxifloxacin 200 mg tablets, 11 antibiotics of the Access group were found physically not available at the time of survey at all the survey sites. They consisted of Access medicines from beta-lactam preparations including amoxicillin injection 250 and 500 mg, ampicillin 1 g injection, phenoxy methyl penicillin tablets, benzathine benzyl penicillin injection, cefazolin 1 g injection, and all preparations of cloxacillin as single ingredient preparations.

Among the antibiotics other than beta lactam antibiotics, chloramphenicol capsules and powder for injection, clindamycin oral liquid, gentamicin 10 mg/mL, 2 mL ampoule, moxifloxacin 200 mg tablet, doxycycline 50 mg capsules, spectinomycin injection and aztreonam injections were not available at any of the surveyed sites (Supplementary Figure S5).

#### Physical availability of essential anti-tuberculosis medicines

Out of the total 28 anti-tuberculosis medicines surveyed in this study, 15 (55%) were found not available at all the survey sites or available at only one site showing the absence of these supplies in routine public and private sector supply chain. Anti-tuberculosis medicines (without duplicates) included the first-line single ingredient and fixed-dose combinations which were 17. In addition to the rest, 11 anti-TB medicines mainly included second-line anti-TB medications that are overlapped for their use in other bacterial infections are grouped as Access, Watch, and Reserve categories. The results showed marked low availability for the anti-TB medicines without duplication represented by anti-TB medicines without duplicates here in this study. Those medicines that were found available belonged to the second-line medications for TB including linezolid the parental preparation of which was available both in private and public sectors except at one public facility.

#### Physical availability of essential antibiotics from the Watch and Reserve groups

Linezolid is a reserved group antibiotic. The powder for the oral dosage form of linezolid was available at all survey sites but was not available at only one public-sector hospital pharmacy. The solid oral dosage form included 400 mg and 600 mg tablets and were available at only three public-sector hospitals and at all private sector pharmacies surveyed, except one (Supplementary Table S8). Aztreonem injections of 500 and 1,000 mg were not found at any survey site.

### Survey 2 results: availability trends

The respondents were also interviewed on the availability trends of essential antibiotic products.

Some of the survey items were available but were reported as in “short supply.” Some anti-TB medicines were described by the respondents as “short in supply.” These were ethambutol + isoniazid tablet 400 mg + 150 mg, ethambutol + isoniazid + pyrazinamide + rifampicin tablet 275 mg + 75 mg + 400 mg + 150 mg, ethambutol + isoniazid + rifampicin tablet 275 mg + 75 mg + 150 mg. Respondents reported that antibiotics including azithromycin 250 mg capsules and 500 mg capsules faced supply shortages during COVID-19 pandemic peak phase (Supplementary Figure S6)

- **Antibiotics not available on survey sites**

- **Not available since a year**

These included anti-TB medicines such as rifampicin oral liquid 20 mg/mL, rifampicin solid oral dosage form 150 mg, rifampicin solid oral dosage form 300 mg, streptomycin injection 1 g, ethambutol 100–400 mg tablet, and ethionamide tablet 250 mg. Other medicinal products that were reported to be “not available since a year” were procaine benzyl penicillin 1 gm powder for injection and ampicillin injection 500 mg.

- **Not available since last 5 years or more**

These essential antibiotics included chloramphenicol capsules 250 mg, chloramphenicol powder for injection 1 g, and clindamycin oral liquid 75 mg/5 mL.

- **Never available**

Some of the essential antibiotics were reported to be never available by the respondents in the availability trend survey, and these included moxifloxacin tablet 200 mg, amikacin 1 g injection, amoxicillin 250 mg injection, amoxicillin 500 mg injection, ampicillin 500 mg injection, ampicillin 1 g injection, benzathine benzylpenicillin 1.44 g benzylpenicillin (2.4 million IU) in 5 mL vial, cefazolin 1 gm injection, chloramphenicol 1 g injection, chloramphenicol 250 mg capsules, clindamycin oral liquid 75 mg/5 mL, cloxacillin capsules 500 mg, cloxacillin powder for injection 500 mg in vial, cloxacillin powder for oral liquid 125 mg/5 mL, cycloserine solid oral dosage form 250 mg, and doxycycline 50 mg capsules.

- **“Do not know”**

A majority of the respondents from public hospitals opted the option “do not know” for essential antibiotics including spectinomycin, aztreonam, phenoxymethylpenicillin and stated that they have never heard about these medicinal products before. Respondents from drug sale outlets stated that they “do not know” or never heard about some of the surveyed medicinal products including aztreonam injection 500 mg, aztreonam 1 g injection, cefazolin 1 g injection, spectinomycin powder for injection 2gm, cloxacillin capsules 500 mg, cloxacillin powder for injection 500 mg in vial, cloxacillin powder for oral liquid 125 mg/5 mL, cycloserine solid oral dosage form 250 mg, gentamycin 10 mg/mL in 2 mL vial, moxifloxacin solid oral dosage form 200 mg, phenoxymethylpenicillin powder for oral liquid 250 mg/5 mL, phenoxymethylpenicillin solid oral dosage form 250 mg, and nitrofurantoin tablet 100 mg.

Apart from the availability of trend options, respondents were also invited to provide any remarks. Some respondents remarked that a few antibiotics under survey were not available at their facility because these were “not in demand.” The antibiotics for which this remark was received included benzyl penicillin injection of 600 mg (1 M IU), kanamycin injection 1 g, amoxicillin+ cloxacillin powder for suspension 125 mg/5 mL, ampicillin 250 mg solid oral dosage form, ampicillin 500 mg solid oral dosage form, meropenem 500 mg injection, cefazolin powder for injection 1 gm, ceftazidime powder for injection 1 g, and vancomycin 500 mg injection. Only one respondent from a public-sector facility shared that “the anti-TB medicines without duplicates are available through specialized centers only and stated that streptomycin 1 g injection was discontinued by the authorities for sale in retail, so it is now not available.”

## Discussion

This is the first study that covers the complete list of essential antibiotics included in the NEML-PK 2018 for availability in public and private sectors in Pakistan. Anti-infective medicines constitute the largest category of NEML-PK 2018 containing a total of 111 generics further expanded to 254 essential antimicrobials (13). It was further divided into sub-categories anthelmintics, antibacterials (antibiotics), anti-fungal medicines, antiviral medicines, and anti-parasitic medicines that were also further categorized into smaller categories (13). The antibacterial sub-category contained 53 generics, expanded to 136 essential antibiotics, out of which 30 essential antibiotics were unregistered, 103 were found registered and 3 medicinal products were duplicated as published in our previous study (13). The remaining 103 registered medicinal products of essential antibiotics were used for field survey to check the availability of these essential antibiotics in public and private sector in the city of Lahore, Pakistan.

A study published in 2014 conducted in 23 countries (low-income countries, lower middle-income countries, and upper middle-income countries) showed that the median availability of essential medicines was suboptimal at 61.5%. The median availability of essential medicines was 40% in the public sector and 78.1% in the private sector. The results of this study are similar to our findings that the availability of essential antibiotics was high in the private sector and lower in the public sector (19). Another study published in October 2022 showed similar results for the availability of essential medicines for asthma and COPD for LMICs. According to the results of this study, the essential medicines used for the treatment of asthma and COPD were largely unavailable and unaffordable (20). However, there are studies on essential medicines and non-communicable diseases medicines but studies on Watch Access and Reserve categories are limited.

The results of a qualitative study based on the response of the selected sample of stakeholders conducted by Atif et al. during May 2018– July 2018 in three cities of Pakistan including Karachi, Islamabad, and Bahawalpur showed that many antimicrobials such as azithromycin, ceftriaxone, quinine, ethambutol, isoniazid were short in supply (21), which are contradicted by our survey results based upon real-time quantitative survey registering the on-spot availability of the survey medicines. The percentage availability of the essential antibiotics recorded by Atif et al. was found adequately available in the current study. The 10 public and private survey sites of Lahore carried out in the current study reveals 60% and 60–100% (250 mg, 500 mg, and 1 g injection of ceftriaxone). This shows the importance of quantitative and on-spot availability survey for more concrete and reliable information. In this study, the availability of ceftriaxone was good (60–100%) as ceftriaxone 250 mg was available at 6 out of 10 sites, ceftriaxone 500 mg injection was available at 7 out of 10 sites, and ceftriaxone 1 g was available at 10 out of 10 survey sites. A complementing data to this study was shown in an availability and pricing study for Lahore conducted in 2016 which documented the availability of ceftriaxone injection as high as 81.8% for lowest price generics in the public sector and for originator brand as 68.8% in the private sector (22).

Similarly, in the current study, the availability of azithromycin in both private as well as public sectors was fairly high (60–80%) as azithromycin 250 mg capsules were present at 8 out of 10 survey sites, azithromycin 500 mg capsule was present at 6 out of 10 survey sites, and azithromycin oral liquid 200 mg/5 mL was available at all private sector retail pharmacies under survey. Despite media reports on hoarding/stockpiling and market shortages of azithromycin period of the COVID-19 pandemic, the availability of the five azithromycin preparations was documented with fairly high availability (60–80%) as recorded in the cumulative values for both sectors at the time of this survey. Similarly, in another field study carried out in 2018, azithromycin’s lowest priced generics were found available in the percentile group of 100% among the surveyed private pharmacy sector (23).

### Availability of anti-infectives and AWaRE classification

Most studies performed in Pakistan employed the WHO/HAI methodology to check the availability of essential medicines including medicines for non-communicable diseases (24–26). This is similar to a study conducted by the Shanti et al. evaluating the availability and affordability of selected essential medicines used for chronic diseases in six low- and middle-income countries (27). The availability of essential antibiotics was very poor in public-sector hospitals, and it was better in private sector retail pharmacies. Similar, results were recorded for 50 essential medicines including six antibiotics in a 2016 study based in Lahore. The mean percent availability for surveyed medicines in the public sector was 6.8% and 35.3% for originator brands (OBs) and lowest price generics (LPGs), respectively, whereas the private sector mean percent availability were 55% and 20.3% for OBs and LPGs, respectively.

#### Low public-sector availability

In the current study, private sector availability was better than the public sector showing marked statistical significance. An average of 23.76 out of 103 medicinal products (ranging from 14 to 28) was observed in the public sector in comparison to the private sector where an average of 59.2 out of 103 medicines were available. The lower public-sector availability points toward lower funding, poor procurement practices with lapses and high stock out periods, issues of disinterest of suppliers in public-sector business resulting in non-participation in public open tenders, the reason being lack of transparency, and delayed procedures and insecurities related to payment. There was overall 30% availability of essential antibiotics at public tertiary care hospitals and overall 66% availability at private sector retail pharmacies. The overall availability of antibiotic was not satisfactory according to the WHO thresh holds (5) with the average of 38.33%, 66.67%, 48.33%, and 11.25% for Access, Watch, Reserve, and antituberculosis medicines.

#### Non-availability of Access group medicines

The most important evidence revealed in the study was that Access group antibiotics had a mode of 0% and median value of 40%. Among the 51 antibiotic preparations surveyed, 17 products were not present at any of the public or private healthcare facilities, which is an alarming situation. Keeping the fact in mind that the products included in the survey were all registered products and the non-availability and shortages of these products call for legal action against companies by the Drug Regulatory Authority of Pakistan (2012) under the Schedule VI of DRAP Act 2012 (The Drug Act 1976, Chapter III, section 30) (28). As shared in the results section, some of the products were remarked by respondents as not in demand at the survey site. Being a tertiary care hospital and its vicinity, this either questions the rationale of inclusion of the products in the NEML or shows the non-availability triggering disinterest of prescriber for that antibiotic resulting in a change in prescribing behaviors. As per the WHO AWaRE classification, the products in Access list must be accessible to population, being evidence-based first-line treatment. Non-availability of first-line treatment in the case of antibiotics leads to the development of antimicrobial resistance by pushing the prescribers to prescribe medicines from the second-line treatment.

#### The essential medicines not available in pharmaceutical market (public and private sector)

This study generates a list of 31 essential antibiotic preparations that were not found at any survey site, out of which 10 are used in the treatment of tuberculosis. The 2018 market survey of private pharmacies in four different regions of Lahore documented the absence of chloramphenicol, cloxacillin, nitrofurantoin, spectinomycin, and cefazolin preparation (23). The same was not found available in the current study except for tablet nitrofurantoin 100 mg. The latter preparation was found in three out of the five private pharmacies but was not available at any of the five public-sector hospitals included in the survey.

Cloxacillin, an anti-staphylococcal penicillin derivative, was found available only in combination with ampicillin in a 1:1 ratio as a powder for suspension for pediatric use in two strengths (125 and 250 mg/5 mL). However, this is not a recommended fixed-dose combination and is listed in the not recommended category in the WHO 2021 Classification of antibiotics (3) due to compromise in the proper dosing of two antibiotics. These two preparations were only found present in private pharmacies accounting for 40% and 50% availability.

Procaine benzylpenicillin is the first-line antibiotic for use in congenital syphilis in children (29) and was not found at any survey sites. This product faced shortage in the United States during 2017 and 2019 and was enlisted in the US FDA drug shortages list, and the prescribers were guided for alternate medication (30).

#### High or uncontrolled Access to Watch group antibiotics

It was clearly observed from the data that Watch and Reserve groups show better accessibility in terms of their availability pattern both in public and private sectors. Apart from one generic aztreonam in the Reserve group and moxifloxacin 200 mg tab in the Watch group, none of the other medicinal products faced 100% non-availability at all survey sites. It is to be noted that aztreonam is now a generic product, and its use has been limited due to its narrow antimicrobial spectrum, cost, and resistance as well as a potential allergenic profile. It was added in WHO EML in 2017 and was withdrawn in 2019 (31). A high consumption of Watch and Reserve group medicines is also documented in other countries with poor regulatory systems advocating the need to restrict Watch group antibiotics and improve Access to Access group antibiotics (32, 33).

The other lowest availability figure for the Watch group was for injection streptomycin 1 g and injection kanamycin 1 g which were only available at two private sector retail pharmacies. The products were part of NEML for their inclusion as the anti-tuberculous agent. The most alarming fact was the 90% availability of injection linezolid 300 mg which is a second-line anti-TB as well as a Reserve group medicine. The oral forms of these antibiotics were predominantly available at all private sector retail pharmacies and were not available in public supplies. Such an availability trend shows the absence of regulatory control on the antibiotic use and a lack or complete absence of awareness of public and private sector facilities and health practices on the aWaRe classification. This shows easy Access to oral Reserve group medicine which is also not expensive and hence prone to misuse on a massive scale in a retail sector of Pakistan, where it is common to dispense antibiotics without prescription (34).

The overall availability of anti-TB medicines was very low in both public and private sectors. This is not a negative indicator as the products are included in the vertical program. However, it was observed from the results of the study that the respondents were not educated enough to give information about availability of anti-TB drugs and that these medicines are supposed to be only available at a specialized center for the treatment of tuberculosis. Only one respondent in the whole survey shared that injection streptomycin 1 g was discontinued by authorities, so it is now not available, and the rest of the products can be accessed from the TB control centers working under the TB control program (35). The strategies and trends from other provinces should also be investigated and documented to rationalize the removal of anti-TB supplies from key public-sector facilities. Apart from the lack of availability and proper information on availability of first-line antituberculous medicine, there was rampant availability of second-line anti-TB medicines which is a devastating picture for perpetuation of antimicrobial resistance (36).

The wide contrast in the percentage availability of antibiotics in the Access category in comparison with and the Watch and Reserve category in a country where antibiotics can still be bought without prescription (36) reflects the misuse and overuse of the latter contributing to the early development of antimicrobial resistance. Some of the antibiotics surveyed in this study are documented for global shortages due to market exits and other reasons. In this study, one of the strengths of benzathine benzyl penicillin, i.e., 2.4 M IU was not available at any survey site, but benzathine benzylpenicillin 1.2 MIU was available at only four private sector pharmacies. Benzathine benzyl penicillin 2.4 MIU injection is used in the prevention of transmission of syphilis from mother to child. The product is of concern for global shortages and stockouts, and the data from Pakistan were missing from the previous global survey covering 95 countries for the period of 2014–2016 (37).

## Conclusion

The study concludes that the reasons for non-availability of medicines, among others are either issues in the supply chain in the public sector or production or supply issues. The non-availability of essential medicines at public-sector facilities is significant and calls for a concrete effort on improvement of procurement methods and removal of potential barriers. It is important that the stakeholders are engaged to devise corrective strategies. A continuous surveillance mechanism through state-funded surveys should be part of the public health policy or the futuristic agenda. The study guides the policy on the restriction of the use of Watch and Reserve medicines, prioritizing Access medicines in registration, import, and manufacturing.

Moreover, the identified medicines that are found not available but are part of global medicine shortage can be good candidates for local manufacturing. Public awareness and education of healthcare professionals on medicine Access issues can result in timely switching to alternates.

The information on the restriction on free Access to Watch and Reserve medicines needs to be advocated through public health campaigns so that people can own and understand the situation.

Courses should be designed for the continuous education of practitioners as well as pharmacists in this area and aWaRe classification concept, and the challenges identified in this study with respect to Access should be made part of the post-graduation as well as undergraduate curriculum. The sale staff of the pharmacies and medical stores should also be educated and be guided to respond properly on the non-available medicines.

This study provides evidence on the unrestricted use of Reserve medicines which calls for policy advice on the use and sale of antibiotics in hospitals as well as at community retail pharmacies.

### Policy recommendations for the future

A rational approach needs to be made to identify the essential antibiotics that are extremely critical. Monitoring of availability of essential medicines through a publicly funded market survey involving a consistent mechanism of identifying and resolving the actual and potential medicine shortages is needed.

The Drug Regulatory Authority of Pakistan must carry out measures for periodic listing of the shortage of medicines through data collected from independent sources on its website, design interventions to correct the situation, and issue a resolution status.

Prescribers should be guided for alternate approaches to treat the patients. The case of Procaine penicillin shortage by the US FDA can be taken as an example (28).

The absence of 20–30% of registered essential antibiotics at retail pharmacies is a serious concern and shows a definitive gap in the capacity of the country to deliver the pharmaceutical needs. A joint consortium of stakeholders is needed to carry out prioritization and consensus on developing and facilitating the means for ensuring the availability of these essential medicines in the country.

National-level demand estimation is needed for the antibiotics that are low in volume. There should be active dissemination of information through a reliable regulatory source on Access to such medicines and means of procurement.

Along with the regulatory measures on prescribing and the restriction on Access to Reserve group medicine, healthcare professionals in both public and private sectors should be trained on the aWaRe concept and rational use of antibiotics. Healthcare facility performance indicators must include antibiotic prescribing patterns, e.g., the use of ACCESS to WATCH Index and ensuring monitoring of antibiotic consumption data.

This is important that the linezolid is a Reserve group medicine, and free Access in the private sector in Pakistan is an opportunity for abuse and misuse, raising the chances of promoting antimicrobial resistance.

## Data availability statement

The datasets presented in this study can be found in online repositories. The names of the repository/repositories and Accession number(s) can be found in the article/Supplementary material.

## Ethics statement

Written informed consent was obtained from the individual(s) for the publication of any potentially identifiable images or data included in this article.

## Author contributions

SR collected the data and written the initial draft. SA performed statistical analysis of data. MU collected the data and finalized the manuscript. HN collected the data and prepared the figures. MC facilitated the data collection and finalized the manuscript. Z-U-DB supervised the study and facilitated in manuscript writing. HR conceived the idea, designed and supervised the study, and finalized the manuscript. All authors contributed to the article and approved the submitted version.
