# Supplementary material for: Availability of Access, Watch, and Reserve groups of essential antibiotics: a cross-sectional survey
Source: Front Public Health. 2024 Jan 4;11:1251434. doi: 10.3389/fpubh.2023.1251434 (PMC10794303; doi:10.3389/fpubh.2023.1251434)
Supplement: Supplementary file 1 [file Data_Sheet_1.docx]

Supplementary Tables:

| **Supplementary Table 1: Demographic details of survey respondents and facilities** | | | | | | | | | | |
| --- | --- | --- | --- | --- | --- | --- | --- | --- | --- | --- |
|  | **Survey site 1** | | **Survey site 2** | | **Survey site 3** | | **Survey site 4** | | **Survey site 5** | |
| **Locality data** | | | | | | | | | | |
| **Facility ID** | **1A** | **1B** | **2A** | **2B** | **3A** | **3B** | **4A** | **4B** | **5A** | **5B** |
| **Facility type** | Public | Private | Public | Private | Public | Private | Public | Private | Public | Private |
| **No. of beds** | 3200 |  | 150 |  | 1200 |  | 164 |  | 1600 |  |
| **No of patients / day** | 6000 |  | 3000 |  | 3000 |  | 1500 |  | 5000 |  |
| **Retail Type** |  | Chain Pharmacy |  | Single Pharmacy |  | Single Pharmacy |  | Single Pharmacy |  | Single Pharmacy |
| **Respondent data** | | | | | | | | | | |
| **Age (Y)** | 35 | 22 | 32 | 28 | 45 | 30 | 45 | 25 | 40 | 19 |
| **Gender** | F | M | M | M | F | M | F | M | F | M |
| **Qualification** | Pharm.D | Intermediate | M.Phil | B.A | B.Pharm | Intermediate | B.Pham | Pharm.D | M.Phil | Owner |
| **Designation** | H.P | Shift Incharge | H.P | Sales Man | H.P | S.S.P  P.O | D.D.C | Pharmacist | H.P | Intermediate |
| **Experience (Y)** | 2 | 10 | 9 | 5 | 15 | 17 | 22 | 1 | 12 | 4 |
| B.A = Bachelor of Arts, H.P = Hospital Pharmacist, S.S.P = Senior Sales Person, P.O = Purchase Officer, D.D.C = Deputy Drug Controller  A= Public Pharmacy, B= Private DSO (Drug Sale Outlet) | | | | | | | | | | |

| **Supplementary Table 2. Availability of 103 essential antibiotic** | | | | |
| --- | --- | --- | --- | --- |
| **Site** | **Sector**  **(Public)** | **Number of products available** | **Sector**  **(Private)** | **Number of products available** |
| 1 | A | 28 | B | 55 |
| 2 | A | 14 | B | 66 |
| 3 | A | 27 | B | 62 |
| 4 | A | 24 | B | 54 |
| 5 | A | 27 | B | 59 |
|  | **Range** | **14-28** | **Range** | **54-66** |
|  | **Average** | **23.76** | **Average** | **59.2** |
|  | **S.D.** | **± 5.19** | **S.D.** | **± 4.45** |

**Supplementary Table 3. Summary of availability status of surveyed products at survey sites**

| **ACCESS GROUP** | | | | | | | | | | | |
| --- | --- | --- | --- | --- | --- | --- | --- | --- | --- | --- | --- |
|  | **Survey sites** | **1A** | **1B** | **2A** | **2B** | **3A** | **3B** | **4A** | **4B** | **5A** | **5B** |
|  | **Available** | 9 | 27 | 5 | 33 | 13 | 29 | 8 | 26 | 10 | 28 |
|  | **Not available** | 42 | 24 | 46 | 17 | 38 | 21 | 43 | 25 | 40 | 22 |
|  | **Available with different specs** | 0 | 0 | 0 | 1 | 0 | 1 | 0 | 0 | 1 | 1 |
| **WATCH GROUP** | | | | | | | | | | | |
|  | **Survey sites** | **1A** | **1B** | **2A** | **2B** | **3A** | **3B** | **4A** | **4B** | **5A** | **5B** |
|  | **Available** | 15 | 21 | 8 | 27 | 12 | 27 | 12 | 25 | 13 | 25 |
|  | **Not available** | 14 | 8 | 21 | 2 | 16 | 2 | 17 | 4 | 14 | 4 |
|  | **Available with different specs** | 0 | 0 | 0 | 0 | 1 | 0 | 0 | 0 | 2 | 0 |
| **RESERVE GROUP** | | | | | | | | | | | |
|  | **Survey sites** | **1A** | **1B** | **2A** | **2B** | **3A** | **3B** | **4A** | **4B** | **5A** | **5B** |
|  | **Available** | 2 | 4 | 1 | 4 | 2 | 4 | 1 | 3 | 1 | 4 |
|  | **Not available** | 4 | 2 | 5 | 2 | 4 | 2 | 5 | 3 | 5 | 2 |
|  | **Available with different specs** | 0 | 0 | 0 | 0 | 0 | 0 | 0 | 0 | 0 | 0 |
| **ANTI TUBERCULAR MEDICINES** | | | | | | | | | | | |
|  | **Survey sites** | **1A** | **1B** | **2A** | **2B** | **3A** | **3B** | **4A** | **4B** | **5A** | **5B** |
|  | **Available** | 2 | 3 | 0 | 2 | 0 | 2 | 3 | 0 | 3 | 2 |
|  | **Not available** | 14 | 14 | 17 | 15 | 17 | 15 | 13 | 17 | 14 | 15 |
|  | **Available with different specs** | 1 | 0 | 0 | 0 | 0 | 0 | 1 | 0 | 0 | 0 |
| **ALL SURVEYED ESSENTIAL ANTIBIOTICS** | | | | | | | | | | | |
|  | **Survey sites** | **1A** | **1B** | **2A** | **2B** | **3A** | **3B** | **4A** | **4B** | **5A** | **5B** |
|  | **Available** | 28 | 55 | 14 | 66 | 27 | 62 | 24 | 54 | 27 | 59 |
|  | **Not available** | 74 | 48 | 89 | 36 | 75 | 40 | 78 | 49 | 73 | 43 |
|  | **Available with different specs** | 1 | 0 | 0 | 1 | 1 | 1 | 1 | 0 | 3 | 1 |

*A= Public Pharmacy, B Private Retail Pharmacies*

| **Supplementary Table 4. Physical availability of essential antibiotics (key access) at survey site** | | | | | | | | | | | | |
| --- | --- | --- | --- | --- | --- | --- | --- | --- | --- | --- | --- | --- |
| **Products description** | | **Physical availability mapping at survey site** | | | | | | | | | | **% Availability** |
| **ID NO.** | **MEDICINE** | **1A** | **1B** | **2A** | **2B** | **3A** | **3B** | **4A** | **4B** | **5A** | **5B** |  |
| **KEY ACCESS ANTI-BIOTICS** | | | | | | | | | | | |  |
| **BETA-LACTAM ANTI-BIOTICS** | | | | | | | | | | | |  |
| 1 | Susp. amoxicillin 125mg/5ml | 2 | 1 | 2 | 1 | 1 | 1 | 1 | 1 | 1 | 1 | 80 |
| 2 | Susp. amoxicillin 250mg/5ml | 1 | 1 | 1 | 1 | 2 | 1 | 2 | 1 | 1 | 1 | 80 |
| 3 | Inj. amoxicillin 250mg | 2 | 2 | 2 | 2 | 2 | 2 | 2 | 2 | 2 | 2 | 0 |
| 4 | Inj. amoxicillin 500mg | 2 | 2 | 2 | 2 | 2 | 2 | 2 | 2 | 1 | 2 | 10 |
| 5 | Cap. amoxicillin 250mg | 2 | 1 | 1 | 1 | 2 | 1 | 2 | 1 | 2 | 1 | 60 |
| 6 | Cap. amoxicillin 500mg | 2 | 1 | 2 | 1 | 2 | 1 | 2 | 1 | 1 | 1 | 60 |
| 7 | Susp. co-amoxiclav 156.25mg | 2 | 1 | 1 | 1 | 2 | 1 | 1 | 1 | 2 | 1 | 70 |
| 8 | Susp. co-amoxiclav 312.5mg | 1 | 1 | 2 | 1 | 1 | 1 | 2 | 1 | 3 | 1 | 80 |
| 9 | Tab. co-amoxiclav 625mg | 1 | 1 | 1 | 1 | 1 | 1 | 1 | 1 | 1 | 1 | 100 |
| 10 | Inj. ampicillin 500mg | 2 | 2 | 2 | 1 | 1 | 2 | 2 | 2 | 2 | 2 | 20 |
| 11 | Inj. ampicillin 1g | 2 | 2 | 2 | 2 | 2 | 2 | 2 | 2 | 2 | 2 | 0 |
| 12 | Cap. ampicillin 250mg | 2 | 1 | 2 | 1 | 2 | 2 | 2 | 2 | 2 | 1 | 30 |
| 13 | Cap. ampicillin 500mg | 2 | 1 | 2 | 1 | 2 | 2 | 2 | 2 | 2 | 1 | 30 |
| 14 | Inj. benzathine benzylpenicillin 1.2million IU | 2 | 1 | 2 | 1 | 2 | 1 | 2 | 2 | 2 | 1 | 40 |
| 15 | Inj. benzathine benzylpenicillin 2.4 million IU | 2 | 2 | 2 | 3 | 2 | 3 | 2 | 2 | 2 | 3 | 30 |
| 16 | Inj. benzylpenicillin 1million IU | 2 | 2 | 2 | 1 | 2 | 1 | 2 | 1 | 2 | 1 | 40 |
| 17 | Susp. phenoxymethylpenicillin 250mg/5ml | 2 | 2 | 2 | 2 | 2 | 2 | 2 | 2 | 2 | 2 | 0 |
| 18 | Tab. phenoxymethylpenicillin 250mg | 2 | 2 | 2 | 2 | 2 | 2 | 2 | 2 | 2 | 2 | 0 |
| 19 | Inj. procaine benzyl penicillin 1g | 2 | 2 | 2 | 2 | 2 | 2 | 2 | 2 | 2 | 2 | 0 |
| 20 | Susp. cefalexin 125mg/5ml | 2 | 1 | 2 | 1 | 2 | 1 | 2 | 1 | 2 | 1 | 50 |
| 21 | Susp. cefalexin 250mg/5ml | 2 | 2 | 2 | 1 | 2 | 1 | 2 | 1 | 2 | 1 | 40 |
| 22 | Cap. cefalexin 250mg | 2 | 2 | 2 | 1 | 2 | 1 | 2 | 1 | 2 | 1 | 40 |
| 23 | Inj. cefazolin 1gm | 2 | 2 | 2 | 2 | 2 | 2 | 2 | 2 | 2 | 2 | 0 |
| 24 | Cap. cloxacillin 500mg | 2 | 2 | 2 | 2 | 2 | 2 | 2 | 2 | 2 | 2 | 0 |
| 25 | Inj. cloxacillin 500mg | 2 | 2 | 2 | 2 | 2 | 2 | 2 | 2 | 2 | 2 | 0 |
| 26 | Susp. cloxacillin 125mg/5ml | 2 | 2 | 2 | 2 | 2 | 2 | 2 | 2 | 2 | 2 | 0 |
| 27 | Susp. cloxacillin + ampicillin 125mg/5ml | 2 | 1 | 2 | 1 | 2 | 1 | 2 | 1 | 2 | 2 | 40 |
| 28 | Susp. cloxacillin + ampicillin 250mg/5ml | 2 | 1 | 2 | 1 | 2 | 1 | 2 | 1 | 2 | 1 | 50 |

| *1= Available* |  | *2=Not Available* |  | *3=Available with slightly different specifications* |
| --- | --- | --- | --- | --- |

| *Availability Grading* |  | *>80%*  *(High)* |  | *50-80%*  *(Fairly High)* |  | *30-49%*  *(<50%)*  *(Low)* |  | *29->0 (<30%)*  *(Very Low)* |  | *0%*  *(Absent or not available)* |
| --- | --- | --- | --- | --- | --- | --- | --- | --- | --- | --- |

*A= Public Pharmacy, B Private DSO (Drug Sale Outlet)*

| **Supplementary Table 5: Physical availability of essential anti-bacterial (access group-other than beta lactam antibiotics) at survey site** | | | | | | | | | | | | |
| --- | --- | --- | --- | --- | --- | --- | --- | --- | --- | --- | --- | --- |
| **Products description** | | **Physical availability mapping at survey site** | | | | | | | | | | |
| **ID NO.** | **MEDICINE** | **1A** | **1B** | **2A** | **2B** | **3A** | **3B** | **4A** | **4B** | **5A** | **5B** | **Availability (%)** |
| **ACCESS GROUP ANTI-BIOTICS-OTHER THAN BETA-LACTAM ANTI-BIOTICS** | | | | | | | | | | | | |
| 29 | Cap. chloramphenicol 250mg | 2 | 2 | 2 | 2 | 2 | 2 | 2 | 2 | 2 | 2 | 0 |
| 30 | Inj. chloramphenicol 1g | 2 | 2 | 2 | 2 | 2 | 2 | 2 | 2 | 2 | 2 | 0 |
| 31 | Inj. amikacin 100mg | 2 | 1 | 2 | 1 | 1 | 1 | 1 | 1 | 2 | 1 | 70 |
| 32 | Inj. amikacin 250mg | 2 | 1 | 2 | 1 | 2 | 1 | 2 | 1 | 1 | 1 | 60 |
| 33 | Caps. clindamycin 150mg | 2 | 1 | 2 | 1 | 2 | 1 | 2 | 1 | 2 | 1 | 50 |
| 34 | Caps. clindamycin 300mg | 1 | 1 | 2 | 1 | 2 | 1 | 2 | 1 | 2 | 1 | 60 |
| 35 | inj. clindamycin 150mg/ml | 2 | 1 | 2 | 1 | 1 | 1 | 2 | 2 | 2 | 1 | 50 |
| 36 | Susp. clindamycin 75mg/5ml | 2 | 2 | 2 | 2 | 2 | 2 | 2 | 2 | 2 | 2 | 0 |
| 37 | Cap. doxycycline 50mg | 2 | 2 | 2 | 2 | 2 | 2 | 2 | 2 | 2 | 2 | 0 |
| 38 | Cap. doxycycline 100mg | 1 | 1 | 2 | 1 | 1 | 1 | 2 | 1 | 1 | 1 | 80 |
| 39 | inj. gentamicin 10mg/ml | 2 | 2 | 2 | 2 | 2 | 2 | 2 | 2 | 2 | 2 | 0 |
| 40 | Inj. gentamicin 40mg/ml | 1 | 1 | 2 | 1 | 1 | 1 | 1 | 1 | 2 | 2 | 70 |
| 41 | Inj. metronidazole 500mg | 1 | 1 | 1 | 1 | 1 | 1 | 1 | 1 | 1 | 1 | 100 |
| 42 | Syrp. metronidazole 200mg/5ml | 2 | 1 | 2 | 1 | 1 | 1 | 1 | 1 | 1 | 1 | 80 |
| 43 | Tab. metronidazole 400mg | 1 | 1 | 2 | 1 | 1 | 1 | 1 | 1 | 1 | 1 | 90 |
| 44 | Tab. nitrofurantoin 100mg | 2 | 2 | 2 | 1 | 2 | 1 | 2 | 1 | 2 | 2 | 30 |
| 45 | Inj. sulfamethoxazole + trimethoprim 80mg+16mg/ml in 10ml ampoule | 2 | 2 | 2 | 2 | 2 | 2 | 2 | 2 | 2 | 2 | 0 |
| 46 | Syrp. sulfamethoxazole + trimethoprim 200mg+40mg/5ml | 2 | 1 | 2 | 1 | 2 | 1 | 2 | 1 | 2 | 1 | 50 |
| 47 | Tab. sulfamethoxazole + trimethoprim 400mg+80mg | 2 | 1 | 2 | 1 | 2 | 1 | 2 | 1 | 2 | 1 | 50 |
| 48 | Tab. sulfamethoxazole + trimethoprim 800mg+160mg | 2 | 1 | 2 | 1 | 1 | 1 | 2 | 1 | 2 | 1 | 60 |
| 49 | Inj. spectinomycin 2g | 2 | 2 | 2 | 2 | 2 | 2 | 2 | 2 | 2 | 2 | 0 |
| 50 | Inj. amikacin 500mg* | 1 | 1 | 2 | 1 | 1 | 1 | 2 | 2 | 2 | 1 | 60 |
| 51 | Inj. amikacin 1g* | 2 | 2 | 2 | 1 | 2 | 2 | 2 | 2 | 2 | 2 | 10 |

| *1= Available* |  | *2=Not Available* |  | *3=Available with slightly different specifications* |
| --- | --- | --- | --- | --- |

| *Availability Grading* |  | *>80%*  *(High)* |  | *50-80%*  *(Fairly High)* |  | *30-49%*  *(<50%)*  *(Low)* |  | *29->0*  *<30%)*  *(Very Low)* |  | *0%*  *(Absent or not available)* |
| --- | --- | --- | --- | --- | --- | --- | --- | --- | --- | --- |

*A= Public Pharmacy, B Private DSO (Drug Sale Outlet)*

| **Supplementary Table 6: Physical availability of essential antibiotic (watch group) at survey sites** | | | | | | | | | | | | |
| --- | --- | --- | --- | --- | --- | --- | --- | --- | --- | --- | --- | --- |
| **Products description** | | **Physical availability mapping at survey site** | | | | | | | | | | |
| **ID NO.** | **MEDICINE** | **1A** | **1B** | **2A** | **2B** | **3A** | **3B** | **4A** | **4B** | **5A** | **5B** | **Availability (%)** |
| **WATCH GROUP ANTIBIOTICS** | | | | | | | | | | | | |
| **BETA-LACTAM ANTI-BIOTICS** | | | | | | | | | | | | |
| 52 | Inj. ceftriaxone 250mg | 2 | 1 | 2 | 1 | 2 | 1 | 2 | 1 | 1 | 1 | 60 |
| 53 | Inj. ceftriaxone 500mg | 2 | 1 | 2 | 1 | 2 | 1 | 1 | 1 | 1 | 1 | 70 |
| 54 | Inj. ceftriaxone 1g | 1 | 1 | 1 | 1 | 1 | 1 | 1 | 1 | 1 | 1 | 100 |
| 55 | Cap. cefixime 400mg | 1 | 1 | 2 | 1 | 1 | 1 | 1 | 1 | 1 | 1 | 90 |
| 56 | Susp. cefixime 100mg/5ml | 1 | 1 | 2 | 1 | 1 | 1 | 1 | 1 | 1 | 1 | 90 |
| 57 | Susp. cefixime 200mg/5ml | 2 | 1 | 2 | 1 | 2 | 1 | 2 | 1 | 2 | 1 | 50 |
| 58 | Inj. piperacillin + tazobactum 2g+250mg | 2 | 2 | 2 | 1 | 1 | 1 | 2 | 2 | 2 | 1 | 40 |
| 59 | Inj. piperacillin + tazobactum 4g+500mg | 1 | 1 | 1 | 1 | 1 | 1 | 1 | 1 | 2 | 1 | 90 |
| 60 | Inj. ceftazidime 250mg | 2 | 2 | 2 | 1 | 2 | 1 | 2 | 1 | 2 | 1 | 40 |
| 61 | Inj. ceftazidime 500mg | 2 | 2 | 2 | 1 | 2 | 1 | 2 | 1 | 2 | 1 | 40 |
| 62 | inj. ceftazidime 1g | 1 | 2 | 2 | 1 | 1 | 1 | 1 | 1 | 2 | 1 | 70 |
| 63 | Inj. meropenum 500mg | 1 | 2 | 1 | 1 | 3 | 1 | 1 | 1 | 1 | 2 | 80 |

| *1= Available* |  | *2=Not Available* |  | *3=Available with slightly different specifications* |
| --- | --- | --- | --- | --- |

| *Availability Grading* |  | *>80%*  *(High)* |  | *50-80%*  *(Fairly High)* |  | *30-49%*  *(<50%)*  *(Low)* |  | *29->0*  *(<30%)*  *(Very Low)* |  | *0%*  *(Absent or not available)* |
| --- | --- | --- | --- | --- | --- | --- | --- | --- | --- | --- |

*A= Public Pharmacy, B Private DSO (Drug Sale Outlet)*

| **Supplementary Table 7: Physical availability of essential antibiotic (watch group other than beta lactam antibiotics) at survey sites** | | | | | | | | | | | | |
| --- | --- | --- | --- | --- | --- | --- | --- | --- | --- | --- | --- | --- |
| **Products description** | | **Physical availability mapping at survey site** | | | | | | | | | | |
| **ID NO.** | **MEDICINE** | **1A** | **1B** | **2A** | **2B** | **3A** | **3B** | **4A** | **4B** | **5A** | **5B** | **Availability (%)** |
| **WATCH GROUP ANTIBIOTICS OTHER THAN BETA-LACTAMS** | | | | | | | | | | | |  |
| 64 | Susp. ciprofloxacin 250mg/5ml | 2 | 1 | 2 | 1 | 2 | 1 | 2 | 1 | 2 | 1 | 50 |
| 65 | Inj. ciprofloxacin 2mg/ml | 1 | 1 | 1 | 1 | 2 | 1 | 1 | 1 | 1 | 1 | 90 |
| 66 | Tab. ciprofloxacin 250mg | 2 | 1 | 2 | 1 | 2 | 1 | 1 | 1 | 2 | 1 | 60 |
| 67 | Tab. ciprofloxacin 500mg | 1 | 1 | 1 | 1 | 1 | 1 | 2 | 1 | 1 | 1 | 90 |
| 68 | Tab. moxifloxacin 200mg | 2 | 2 | 2 | 2 | 2 | 2 | 2 | 2 | 2 | 2 | 0 |
| 69 | Tab. moxifloxacin 400mg | 1 | 1 | 2 | 1 | 1 | 1 | 2 | 1 | 1 | 1 | 80 |
| 70 | Cap. azithromycin 250mg | 2 | 1 | 2 | 1 | 1 | 1 | 1 | 1 | 1 | 1 | 80 |
| 71 | Cap. azithromycin 500mg | 1 | 1 | 2 | 1 | 2 | 1 | 2 | 1 | 2 | 1 | 60 |
| 72 | Susp. azithromycin 200mg/5ml | 2 | 1 | 2 | 1 | 2 | 1 | 2 | 1 | 3 | 1 | 60 |
| 73 | Susp. clarithromycin 125mg/5ml | 1 | 1 | 2 | 1 | 2 | 1 | 2 | 1 | 3 | 1 | 70 |
| 74 | Tab. clarithromycin 500mg | 1 | 1 | 2 | 1 | 1 | 1 | 2 | 1 | 2 | 1 | 70 |
| 75 | Inj. vancomycin 500mg | 1 | 2 | 1 | 1 | 1 | 1 | 1 | 1 | 2 | 2 | 70 |
| 76 | Inj. kanamycin 1g** | 2 | 1 | 2 | 2 | 2 | 2 | 2 | 2 | 2 | 1 | 20 |
| 77 | Tab. levofloxacin 250mg** | 1 | 1 | 1 | 1 | 2 | 1 | 1 | 1 | 1 | 1 | 90 |
| 78 | Tab. levofloxacin 500mg** | 1 | 1 | 1 | 1 | 1 | 1 | 2 | 1 | 1 | 1 | 90 |
| 79 | Tab. levofloxacin 750mg** | 2 | 1 | 2 | 1 | 2 | 1 | 2 | 1 | 1 | 1 | 60 |
| 80 | Inj. streptomycin 1g** | 2 | 2 | 2 | 1 | 2 | 1 | 2 | 2 | 2 | 2 | 20 |

***Also included in the NEML list of anti-tuberculous medicines*

| *1= Available* |  | *2=Not Available* |  | *3=Available with slightly different specifications* |
| --- | --- | --- | --- | --- |

| *Availability Grading* |  | *>80%*  *(High)* |  | *50-80%*  *(Fairly High)* |  | *30-49%*  *(<50%)*  *(Low)* |  | *29->0*  *(<30%)*  *(Very Low)* |  | *0%*  *(Absent or not available)* |
| --- | --- | --- | --- | --- | --- | --- | --- | --- | --- | --- |

*A= Public Pharmacy, B Private DSO (Drug Sale Outlet)*

| **Supplementary Table 8: Physical availability of essential antibiotic (Reserve group with Anti-TB medicines) at survey sites** | | | | | | | | | | | | |
| --- | --- | --- | --- | --- | --- | --- | --- | --- | --- | --- | --- | --- |
| **Products description** | | **Physical availability mapping at survey site** | | | | | | | | | | |
| **ID NO.** | **MEDICINE** | **1A** | **1B** | **2A** | **2B** | **3A** | **3B** | **4A** | **4B** | **5A** | **5B** | **Availability (%)** |
| **RESERVE GROUP ANTIBIOTICS WITH ANTI-TUBERCULOSIS** | | | | | | | | | | | | |
| 81 | Inj. aztreonam 500mg | 2 | 2 | 2 | 2 | 2 | 2 | 2 | 2 | 2 | 2 | 0 |
| 82 | Inj. aztreonam 1g | 2 | 2 | 2 | 2 | 2 | 2 | 2 | 2 | 2 | 2 | 0 |
| 83 | Inj. linezolid 2mg/ml in 300ml bag*** | 1 | 1 | 1 | 1 | 1 | 1 | 1 | 1 | 2 | 1 | 90 |
| 84 | Susp. linezolid 100mg/5ml*** | 2 | 1 | 2 | 1 | 2 | 1 | 2 | 1 | 2 | 1 | 50 |
| 85 | Tab. linezolid 400mg*** | 2 | 1 | 2 | 1 | 2 | 1 | 2 | 2 | 1 | 1 | 50 |
| 86 | Tab. linezolid 600mg*** | 1 | 1 | 2 | 1 | 1 | 1 | 2 | 1 | 2 | 1 | 70 |

****Also included in the NEML list of anti-tuberculous medicines*

| *1= Available* |  | *2=Not Available* |  | *3=Available with slightly different specifications* |
| --- | --- | --- | --- | --- |

| *Availability Grading* |  | *>80%*  *(High)* |  | *50-80%*  *(Fairly High)* |  | *30-49%*  *(<50%)*  *(Low)* |  | *29->0*  *(<30%)*  *(Very Low)* |  | *0%*  *(Absent or not available)* |
| --- | --- | --- | --- | --- | --- | --- | --- | --- | --- | --- |

*A= Public Pharmacy, B Private DSO (Drug Sale Outlet)*

| **Supplementary Table 9: Physical availability of essential antibiotic (Anti-TB medicines) at survey sites** | | | | | | | | | | | | |
| --- | --- | --- | --- | --- | --- | --- | --- | --- | --- | --- | --- | --- |
| **Products description** | | **Physical availability mapping at survey site** | | | | | | | | | | |
| **ID NO.** | **MEDICINE** | **1A** | **1B** | **2A** | **2B** | **3A** | **3B** | **4A** | **4B** | **5A** | **5B** | **Availability (%)** |
| **6.2.3 ANTI-TB MEDICINES** | | | | | | | | | | | | |
| 87 | Tab. ethambutol 100mg-400mg | 2 | 2 | 2 | 2 | 2 | 2 | 2 | 2 | 1 | 2 | 10 |
| 88 | Tab. ethambutol+isoniazid 400mg+150mg | 2 | 2 | 2 | 2 | 2 | 2 | 2 | 2 | 2 | 2 | 0 |
| 89 | Tab. ethambutol+isoniazid+ pyrazinamide+rifampicin 275mg+75mg+400mg+150mg | 1 | 1 | 2 | 1 | 2 | 1 | 1 | 2 | 1 | 1 | 70 |
| 90 | Tab. ethambutol+isoniazid+ rifampicin 75mg+75mg+150mg | 3 | 1 | 2 | 1 | 2 | 1 | 2 | 2 | 2 | 1 | 40 |
| 91 | Syrp. isoniazid 50mg/5ml | 2 | 2 | 2 | 2 | 2 | 2 | 2 | 2 | 2 | 2 | 0 |
| 92 | Tab. isoniazid 100mg-300mg | 2 | 2 | 2 | 2 | 2 | 2 | 2 | 2 | 2 | 2 | 0 |
| 93 | Tab. scored isoniazid 50mg | 2 | 2 | 2 | 2 | 2 | 2 | 2 | 2 | 2 | 2 | 0 |
| 94 | Tab. isoniazid + pyrazinamide + rifampicin 75mg+400mg+150mg | 2 | 2 | 2 | 2 | 2 | 2 | 3 | 2 | 2 | 2 | 10 |
| 95 | Tab. isoniazid + pyrazinamide + rifampicin 150mg+500mg+150mg | 2 | 2 | 2 | 2 | 2 | 2 | 2 | 2 | 2 | 2 | 0 |
| 96 | Tab. isoniazid+rifampicin 75mg+150mg | 1 | 2 | 2 | 2 | 2 | 2 | 1 | 2 | 1 | 2 | 30 |
| 97 | Tab. isoniazid + rifampicin 150mg+300mg | 2 | 2 | 2 | 2 | 2 | 2 | 1 | 2 | 2 | 2 | 10 |
| 98 | Syrp. rifampicin 20mg/ml | 2 | 1 | 2 | 2 | 2 | 2 | 2 | 2 | 2 | 2 | 10 |
| 99 | Tab. rifampicin 150mg | 2 | 2 | 2 | 2 | 2 | 2 | 2 | 2 | 2 | 2 | 0 |
| 100 | Tab. rifampicin 300mg | 2 | 2 | 2 | 2 | 2 | 2 | 2 | 2 | 2 | 2 | 0 |
| 101 | Cap. cycloserine 250mg | 2 | 2 | 2 | 2 | 2 | 2 | 2 | 2 | 2 | 2 | 0 |
| 102 | Tab. ethionamide 125mg | 2 | 2 | 2 | 2 | 2 | 2 | 2 | 2 | 2 | 2 | 0 |
| 103 | Tab. ethionamide 250mg | 2 | 2 | 2 | 2 | 2 | 2 | 2 | 2 | 2 | 2 | 0 |
|  | Inj. streptomycin 1g** | 2 | 2 | 2 | 1 | 2 | 1 | 2 | 2 | 2 | 2 | 20 |
|  | Inj. amikacin 500mg* | 1 | 1 | 2 | 1 | 1 | 1 | 2 | 2 | 2 | 1 | 60 |
|  | Inj. amikacin 1g* | 2 | 2 | 2 | 1 | 2 | 2 | 2 | 2 | 2 | 2 | 10 |
|  | Inj. kanamycin 1g** | 2 | 1 | 2 | 2 | 2 | 2 | 2 | 2 | 2 | 1 | 20 |
|  | Tab. levofloxacin 250mg** | 1 | 1 | 1 | 1 | 2 | 1 | 1 | 1 | 1 | 1 | 90 |
|  | Tab. levofloxacin 500mg** | 1 | 1 | 1 | 1 | 1 | 1 | 2 | 1 | 1 | 1 | 90 |
|  | Tab. levofloxacin 750mg** | 2 | 1 | 2 | 1 | 2 | 1 | 2 | 1 | 1 | 1 | 60 |
|  | Inj. linezolid 2mg/ml in 300ml bag*** | 1 | 1 | 1 | 1 | 1 | 1 | 1 | 1 | 2 | 1 | 90 |
|  | Susp. linezolid 100mg/5ml*** | 2 | 1 | 2 | 1 | 2 | 1 | 2 | 1 | 2 | 1 | 50 |
|  | Tab. linezolid 400mg*** | 2 | 1 | 2 | 1 | 2 | 1 | 2 | 2 | 1 | 1 | 50 |
|  | Tab. linezolid 600mg*** | 1 | 1 | 2 | 1 | 1 | 1 | 2 | 1 | 2 | 1 | 70 |

| *1= Available* |  | *2=Not Available* |  | *3=Available with slightly different specifications* |
| --- | --- | --- | --- | --- |

| *Availability Grading* |  | *>80%*  *(High)* |  | *50-80%*  *(Fairly High)* |  | *30-49%*  *(<50%)*  *(Low)* |  | *29->0*  *(<30%)*  *(Very Low)* |  | *0%*  *(Absent or not available)* |
| --- | --- | --- | --- | --- | --- | --- | --- | --- | --- | --- |

*A= Public Pharmacy, B Private DSO (Drug Sale Outlet),* **Access group ** Watch group ***Reserve group*

**Supplementary Table 10. Summary of Physical Availability from 103 surveyed essential antibiotic**

| **AVAILABILITY STATUS** | **Percentage (%)** |
| --- | --- |
| Medicinal products available at tertiary care hospital with slightly different specifications | 5.82 |
| Medicinal products available at one tertiary care hospital | 18.44 |
| Medicinal products not available at any tertiary care hospital | 21.35 |
| Medicinal products not available at any pharmacy/medical store | 3.88 |
| Medicinal products available at all survey sites | 2.91 |
| Medicinal products not available at any survey site | 30.09 |
| Medicinal products available at all pharmacies/medical stores | 40.77 |
| Medicinal products available at all tertiary care hospitals | 2.91 |
| Medicinal products not available in one tertiary care hospital | 9.70 |
| Medicinal products available at only one pharmacy/medical store | 2.91 |
| Medicinal products not available at one pharmacy/medical store | 12.62 |
