# Supplementary figures and images for: Availability of Access, Watch, and Reserve groups of essential antibiotics: a cross-sectional survey
Source: Front Public Health. 2024 Jan 4;11:1251434. doi: 10.3389/fpubh.2023.1251434 (PMC10794303; doi:10.3389/fpubh.2023.1251434)

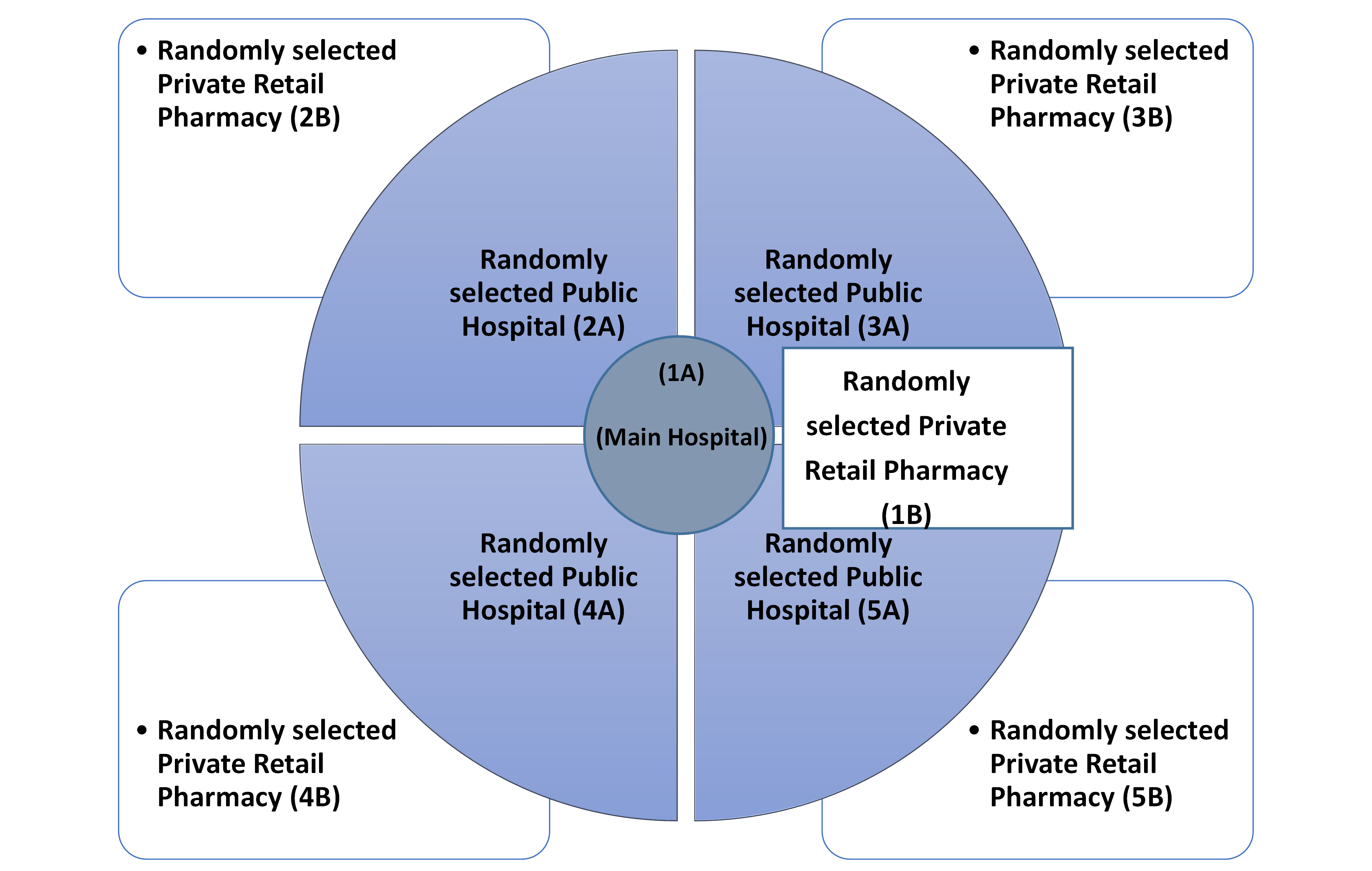

Supplement: Supplementary file 2 [file Image_1.TIF]

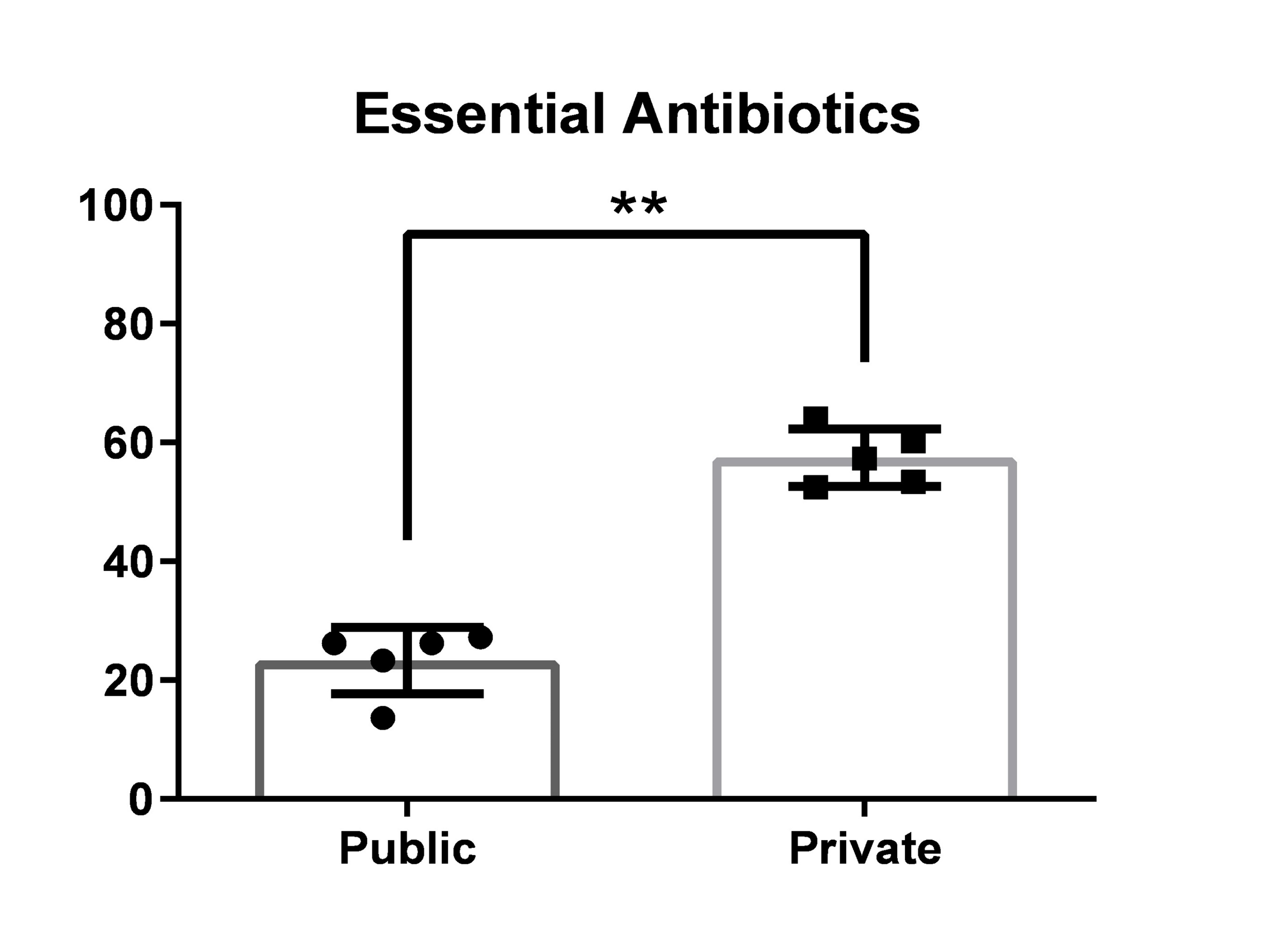

Supplement: Supplementary file 3 [file Image_2.TIF]

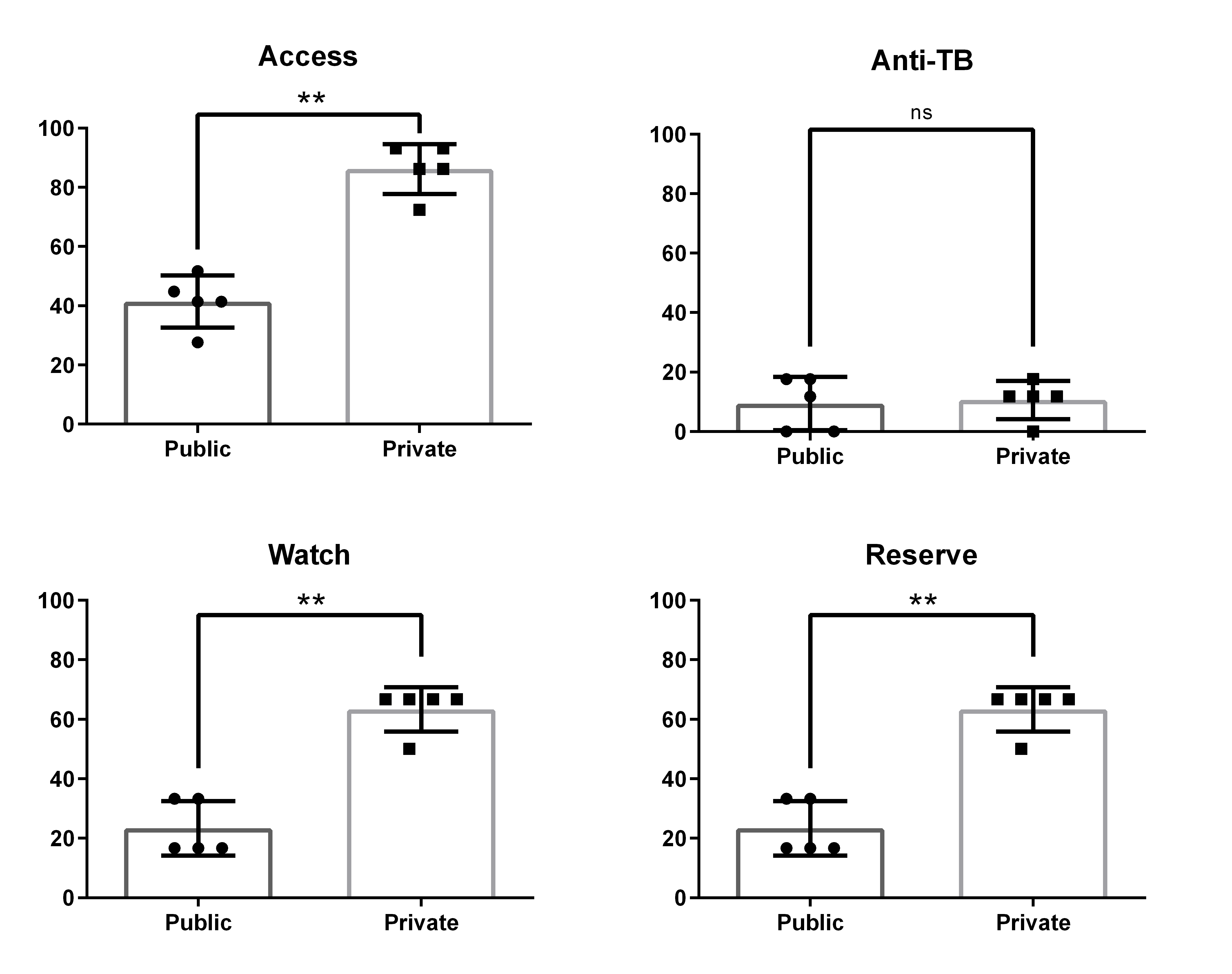

Supplement: Supplementary file 4 [file Image_3.TIF]

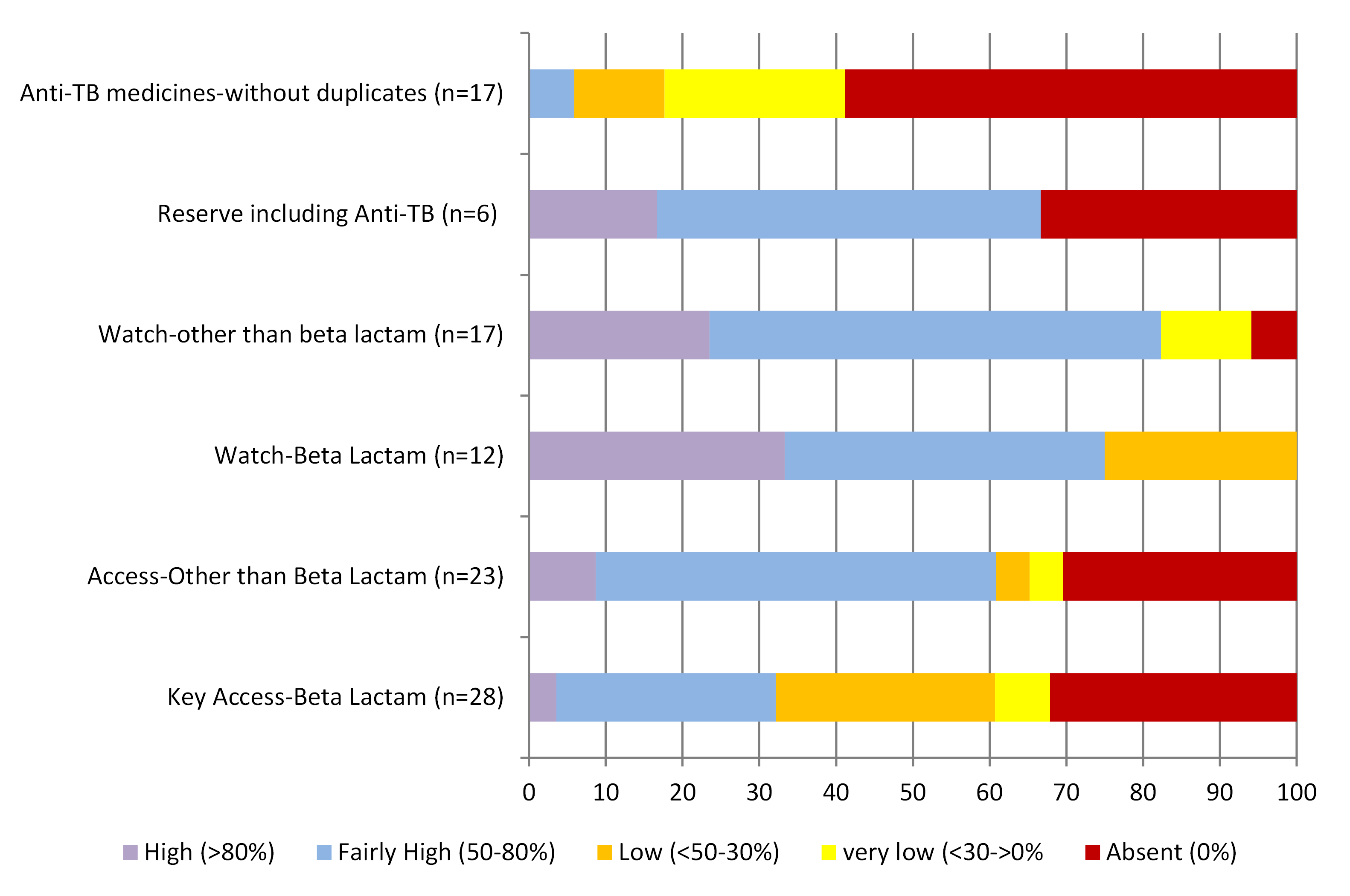

Supplement: Supplementary file 5 [file Image_4.TIF]

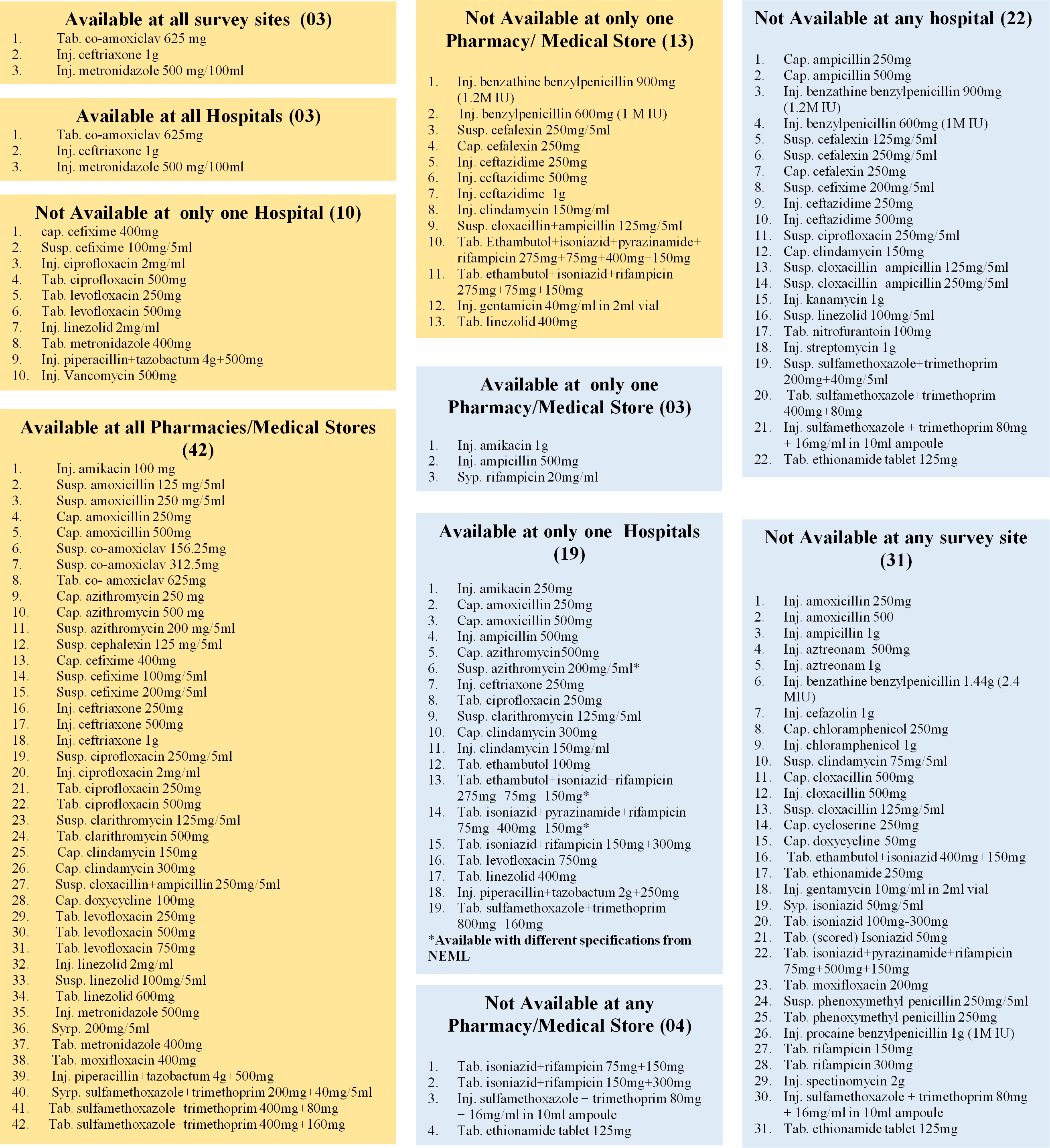

Supplement: Supplementary file 6 [file Image_5.TIF]

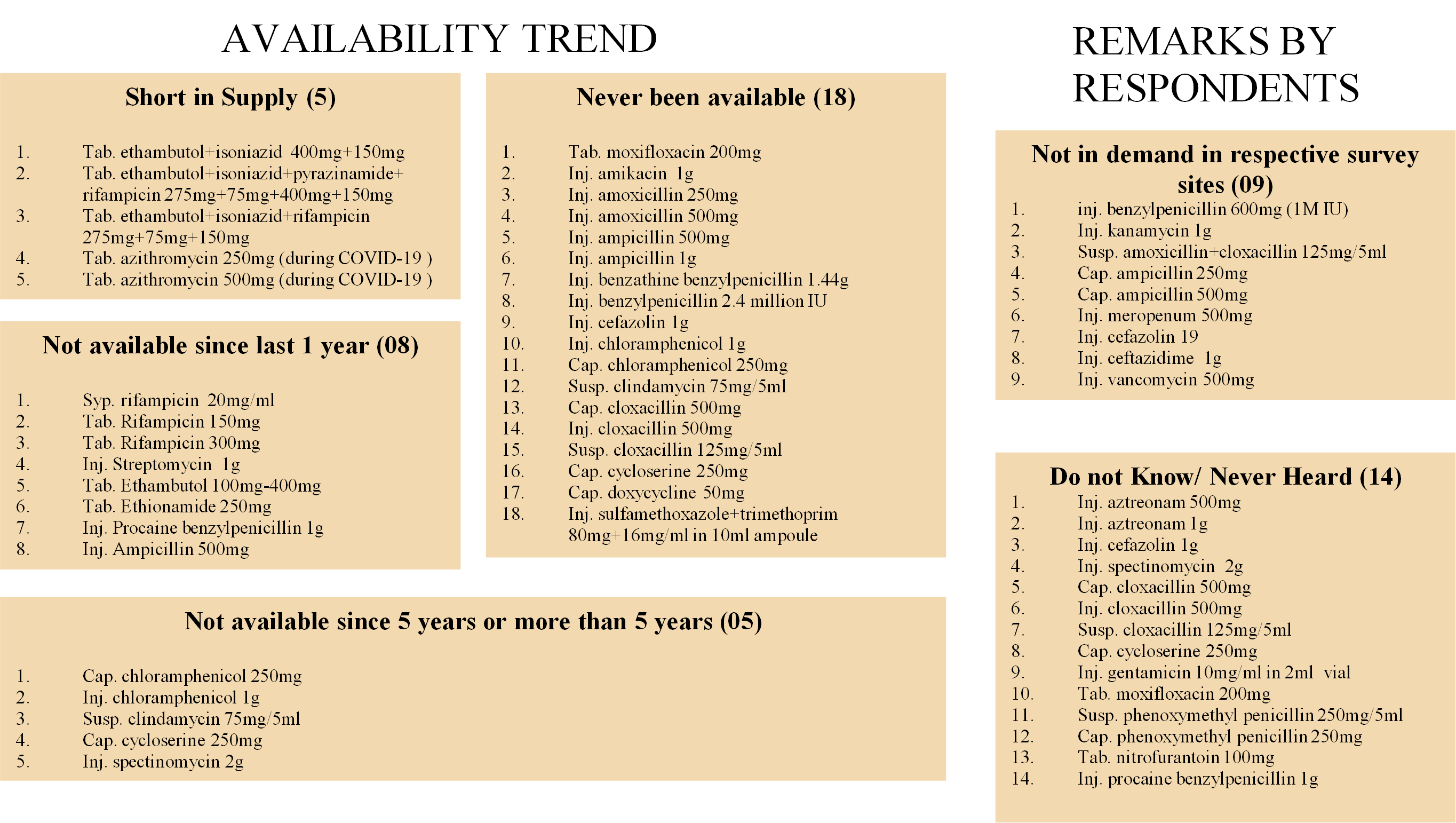

Supplement: Supplementary file 7 [file Image_6.TIF]
